# Logic Gates Based on DNA Aptamers

**DOI:** 10.3390/ph13110417

**Published:** 2020-11-23

**Authors:** Mariia Andrianova, Alexander Kuznetsov

**Affiliations:** Scientific-Manufacturing Complex Technological Centre, 1–7 Shokin Square, Zelenograd, 124498 Moscow, Russia; kae@tcen.ru

**Keywords:** aptamer, logic gate, sensor, biosensor

## Abstract

DNA bio-computing is an emerging trend in modern science that is based on interactions among biomolecules. Special types of DNAs are aptamers that are capable of selectively forming complexes with target compounds. This review is devoted to a discussion of logic gates based on aptamers for the purposes of medicine and analytical chemistry. The review considers different approaches to the creation of logic gates and identifies the general algorithms of their creation, as well as describes the methods of obtaining an output signal which can be divided into optical and electrochemical. Aptameric logic gates based on DNA origami and DNA nanorobots are also shown. The information presented in this article can be useful when creating new logic gates using existing aptamers and aptamers that will be selected in the future.

**List of Contents**

**1.**  **Introduction**
**Main Text**
**2.**  **Optical Detection**
  **2.1.**  **Fluorescent Output in Solution**
  **2.2.**  **The Colorimetric Output in the Solution Due to the Catalytic Labels**
  **2.3.**  **Logical Gates Based on Gold Nanoparticles**
    **2.3.1.**  **Logic Gates Based on Covalently Modified Gold Nanoparticles**
**Lateral Flow Strip Biosensors**
**Mesoporous Silicon**
    **2.3.2.**  **Logic Gates Based on Covalently Modified Gold Nanoparticles**
**Hydrogels**
  **2.4.**  **Logical Gates Based on Graphene Oxide**
**3.**  **Electrochemical Detection**
  **3.1.**  **Two-/Three-Electrode Cells**
    **3.1.1.**  **Electroactive Compounds**
    **3.1.2.**  **Catalytic Labels**
  **3.2.**  **Biofuel Cells**
  **3.3.**  **Other**
**4.**  **Cell-Based Logic Gates**
**5.**  **Logic Gates Based on DNA Origami**
**6.**  **Conclusions**

## 1. Introduction

In the modern scientific world, a popular direction is the development of alternative approaches to computing, in particular, based on interactions among biomolecules—bio-computing. Since the capabilities of silicon technology are limited by the size of the components and the speed of their operation [1], then the transition to biocomputers will potentially overcome some of the limitations and create a new type of technology for storing and processing a large amount of information. Bio-computing implies the construction of computations and logical operations based on biocomponents: DNA, RNA, enzymes and cells [2]. Thus, a lot of work has already been done to create logical operations involving DNA [3,4], RNA [5], DNAzymes [6,7,8] and enzymes [9,10]. These systems can perform basic arithmetic operations, such as addition and subtraction [11,12], calculate the square root [13], play logic games [14,15] and simulate keyboard lock [16,17]. There are reviews of works on the use of biocomputer logical calculations at the level of one cell: targeted drug delivery and visualization [18].

A particular case of DNA bio-computing is the use of aptamers and their direct ability to selectively form a complex with targets.

Aptamers are artificially selected functional DNA or RNA oligonucleotides as well as peptides that selectively bind to target compounds. The targets are proteins, low and high molecular weight compounds, metal ions, and cells. To narrow the scope of consideration, this review considers DNA aptamers. DNA aptamers have several unique characteristics: relative ease of their obtention via the SELEX (Systematic Evolution of Ligands by EXponential enrichment) method [19,20]; synthetic nature, allowing easy and economic synthesis with high reproducibility and purity; the ability to obtain chemically modified aptamers. Moreover, since such aptamers are nucleic acids, they also have programmability, predictability, and high information storage density due to the principle of complementary base pairing [21]. Thus, DNA aptamers can be considered to be unique biomimetic receptors with properties suitable for constructing logic gates. They can analyze a series of inputs and decide whether or not to produce an output based on the internal logic of the system of which they are a part.

**Figure 1:**
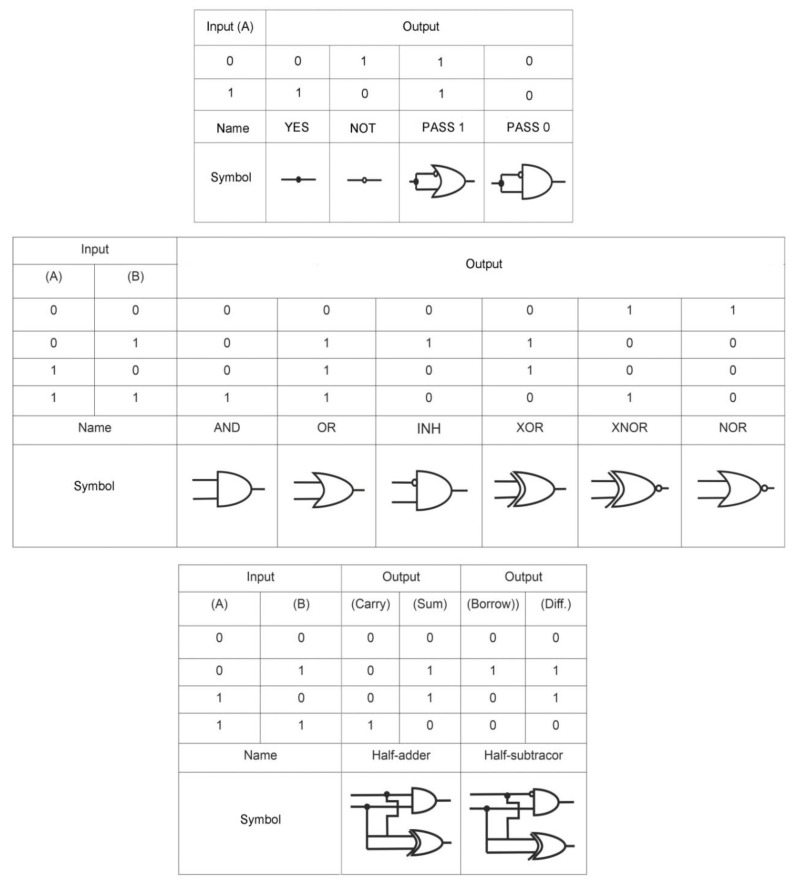
Logic gates: inputs **A**, **B** (0, 1), outputs (0, 1) and symbols.

By definition, a logic gate is a basic element of a digital circuit that performs an elementary conversion of input signals into a logic output signal. In binary logic, these are binary switchers that have input conditions (0 or 1) that determine the output state (0 or 1). Figure 1 is considered a set of basic logic gates using Boolean computation and performing a set of operations via silicon computer. By analogy, these operations can be extended to bio-computing with the participation of aptamers.

For example, an AND gate has two inputs, A and B; output signal 1 only occurs when A and B are present together (1, 1); the presence of only A (1, 0) or B (0, 1) does not lead to the occurrence of the output signal, which is taken as 0. For the logic gate OR, the output signal 1 is formed when there is one A (1, 0), one B (0, 1), or both A and B (1, 1) in the system. The output of the logic gate XOR is assumed to be 1 when A (1, 0) or B (0, 1) are present, but not when both are present. Other basic logic gates can be interpreted in the same way. In this case, under the conditions of aptameric bio-computing, biomolecules (for example, targets) act as A and B, and optical (fluorescent, colorimetric) or electrochemical signals as an output signal. In the case of an output signal, a threshold value is set, above which the signal is taken as 1, and below it as 0. Thus, the measurement results are simplified: either “yes” or “no”, or analytes in the samples are “present” or “not present”.

From the logic point of view, a *biosensor* based on aptamers can be considered to be an elementary logic gate YES. The presence of a target in the system results in an output signal. The detection limit can be considered to be the threshold value for the appearance of a signal and assignment of output 1 to it and the detection range as a window for a stable output signal 1. The difference between a sensor and a logic gate is the ability to create a set of gates that operate according to the internal law of logic and use several inputs. 

In addition to logic gates, there are *logic circuits* involving aptamers, which are not considered in detail in this review. In logic circuits, an aptamer is part of a general circuit in which there is no simultaneous analysis of several aptamer targets and generation of a summed response, unlike logic gates. In some schemes, the functional action of the aptamer (formation of a complex with the target) leads to the generation of further logical operations and to the emergence of a subsequent result of the action of the entire scheme, i.e., a downstream cascade mechanism is realized relative to the action of the aptamer [22,23,24,25,26,27]. In other logic circuits, certain operations can lead to the formation/decay of a complex between the aptamer and the target—an upstream cascade mechanism [28]. Sometimes both types of cascades occur in the same scheme with the participation of one target but different aptamers [29,30]. The logic circuit can accurately detect the local concentration of thrombin, and when it is excessively high, the clotting inhibitor is automatically released by the circuit module with a controlled concentration of internal components [30]. This work uses two aptamers against thrombin. In [27], a DNA association driven by the target adenosine triphosphate (ATP) was shown to initiate enzyme-free cyclic hairpin assembly. It is also known that the formation of a complex between cocaine and cleaved parts of its aptamer, each part containing a cofactor/enzyme or enzyme/enzyme, led to the spatial assembly of the biocatalytic system [22]. There are publications in which switching an aptamer from a complementary sequence to a target led to a further cascade of reactions [30,31].

According to the analysis of the literature, it can be concluded that the existing logic gates based on aptamers can be divided into two categories. The first category is those that use a set of targets, the analysis of which is of practical importance; in other words, aptamer targets are found in the same environment. In this case, cell membrane markers and proteins [32,33,34,35,36,37,38,39,40], enantiomers [41], structurally similar compounds [42] and antibiotics [43] are inputs. The second category of logic gates is based on known aptamers for targets such as thrombin [44,45], lysozyme [46], ATP [47], adenosine [47], adenosine 5′-monophosphate [48] and cocaine [49,50]. The combined use of these targets as inputs to logic gates is probably not of practical importance for analytical chemistry. At the same time, the proposed approaches in the future can be useful for creating practical logic gates and operations. In the implementation of logic gates of the second category, special attention is paid to platforms and sensitive elements (graphene oxide, electrodes, gold nanoparticles, etc.) to receive a signal from interaction with targets.

The main aptamer strategies in the implementation of logic gates:

- Formation of a complex of an aptamer with a target with a change in spatial configuration; sandwich formation (one target versus two aptamers);
- Switching an aptamer from dsDNA (hairpin, complementary DNA) to a complex with a target;
- The use of split aptamers into two parts and the formation of a complex of the split parts with the target;
- The use of hybrid DNA based on cleaved and combined parts of aptamers;
- Use of a bifunctional aptamer containing two aptamers.

The mechanism of action of logic gates is often based on switching of the aptamer structure [51]: the addition of a target leads to a change in the spatial structure of the aptamer and, for example, the dissociation of the DNA duplex. Thus, the aptamer switches from one structure to another [52]. The design of complementary regions is very important in this case, since there is an energy balance between hybridization and dehybridization. Maintaining this balance results in logic implementation with the least possible interference and baseline noise. At the same time, all DNA sequences participating in logic gates can impact on each other (conformationally or be partially complementary) as a result of which the efficiency of their interaction with targets can decrease. This once again confirms the special importance of the DNA design of logic gates.

On the other hand, it is interesting to use bifunctional aptamers, parts of cleaved aptamers and cleaved parts of aptamers in the composition of hybrid DNA. Most often, such aptamers are assembled into a complex in the presence of a target, which can be used to diagnose several markers simultaneously: their joint presence or the presence of at least one of them. Logic gates are based on split anti-ATP [53,54], adenosine [55], thrombin [54,56] and cocaine [55] aptamers. The possibility of using other split aptamers is questionable and requires research. 

In addition, it is possible to distinguish common DNA strategies that frequently occur in the organization of aptameric logic gates:

- Cascade: the cascade effect is realized when the reactions occurring in the system are interconnected. The products of one reaction initiate the start of a new reaction. Examples of a cascade in aptameric logic can be found in [32,34,35,40,57,58];
- Toehold-mediated strand displacement: This strategy is the most common in the organization of DNA logic and bio-computing in general [59]. It implies the replacement of one complementary sequence in dsDNA with another, which initially binds to a free region—a toehold. The substitution reaction is energetically preferred [60]. This strategy is often encountered when organizing cascade reactions [32,34,35,40,57,58].

The first molecular logic gate that used aptamers to define input targets was developed by Jose et al. [61]. The RNA aptamer against flavin mononucleotide and theophylline were combined with a hammerhead-shaped self-cleaving ribozyme resulting in an AND logic gate. The first inclusion of a DNA aptamer in logic was described by Kolpashchikov et al. [28]. The authors used a malachite green aptamer [62] in the NAND and NOT gates and an aptamer on Taq DNA polymerase [63] in the AND gate. In this work, aptamers took an indirect part in the logic, in which the main gates were cleaving DNAzymes, and the input data were ssDNAs, which allosterically regulated their activity.

In addition to elementary gates based on aptamers with two input data (iORi, iANDi, iXORi, iNORi, iINHIBITi, iNANDi, iNOTi, iCNOTi), gates with three and four input data (iANDiANDi, iANDiORi, iANDiINHIBITHI, iNANDi iANDiINHIBITiNANDi, iORiINHIBITiNANDi, iANDiORiINHIBITiNANDi, iINHIBITiANDi, iINHIBITiANDiXORi) were fabricated (Supplementary Materials Table S1). Wherein, as a rule, only two inputs were assigned to aptamer targets, and the remaining inputs were more often ssDNA including the aptamers themselves. There were also implemented half-adder and half-subtractor based on aptamers [37,64]. The operation time of the logic gates from the moment of entering the input data to the moment of receiving the output signal averaged 60–120 min (Supplementary Materials Table S1). Separately, it is worth noting the variety of approaches to the creation of logic gates based on aptamers. It is directly reflected in the organization of the experiment: the design of DNA constructs and the use of various signal transducers. The main difficulties in creating logic are the basic noise associated with the functioning of signal converters and interference caused by cross-reactions in the ssDNA system with each other.

Conventional electronic circuits can perform multi-level operations; however, this capability is rarely realized by aptamer-based logic gates. In addition, the question of how to combine biomolecular computation and silicon-based electrical circuits remains a key issue in bioelectronics. Therefore, at the moment, the main areas of application of logic gates based on aptamers are diagnostic and analytical tasks. For diagnostic purposes, such gates can be used as a sensor platform for working with a complex environment: simultaneous determination of a set of targets (joint presence or absence, mutual exclusion, etc.). This approach allows for more complete and complex diagnostics, accurate diagnosis, or targeted drug delivery in the presence of disease indicators. Analytical tasks involve analyzing a multi-component environment: drugs production, food, etc.

This review is devoted to logic gates implemented based on DNA aptamers against targets. DNA sequences that bind metal ions (metal-mediated base paring) will not be considered. Structurally, the overview consists of parts, divided according to the principle of detecting the output signal and working objects (cells, DNA origami). Each beginning of a new part contains summarized information on this section with the identification of main patterns and a brief description of the gates known to date. The overview also contains pictures of some gates to have a better idea of how they work.

## 2. Optical Detection

The use of an optical signal as the output data of logic gates with aptamers is quite widespread. Within the framework of logic, the optical signal can be divided into colorimetric [38,41,54,55,58,65,66,67,68,69,70,71,72] and fluorescent [38,39,57,73,74,75,76,77,78,79].

In turn, the colorimetric signal is formed due to the catalytic label that catalyzes the reaction with the formation of a colored product or due to the presence of gold nanoparticles (discussed further in the subsection), and the color of the solution which is determined by the size of the nanoparticles, the distance between them and their shape (plasmon resonance effect). As a catalytical label a peroxidase-like DNAzyme is used [58,65] which is released from an inactive state as a result of the interaction of aptamers with targets. 

The formation of a fluorescent signal is based on the use of fluorescent labels (fluorophore, quantum dots, silver nanoclusters) and quenchers located close to each other. As a result of the action of the logic, a spatial separation of the label and the quencher occurs and the appearance of a fluorescent signal [64,73,76,77,79]. According to the construction of logic gates, it is possible to distinguish those systems that occur in solution and systems that include the participation of the third component—the carriers. In solution, the fluorescence of the label is quenched by a chemical quencher [73,79]. In the case of carriers, such as graphene oxide [80,81] and gold nanoparticles [74], fluorescence labels which are located close to their surface are quenched due to the resonance energy transfer. 

### 2.1. Fluorescent Output in Solution

This section discusses the classical scheme with hybridized dsDNA with fluorescent label fluorescein and quenchers [73]; shows the use of DNA-modified magnetic microparticles and quantum dots for the determination of prions [39]; suggests the use of silver nanoclusters and peroxidase-like DNAzyme with hemin in composition of one DNA sequence for platelet-derived growth factor (PDGF) determination [79]. In addition, a logic gate was built with the participation of microparticles with a smart coating containing aptamers which reacts to the presence of a target [82].

Construction of logic OR and AND gates based on aptamers is described in [73]. It uses aptamers for thrombin [44] and adenosine [47] in one ssDNA connected by a linker in 11 bp. When the AND logic was implemented, the base labeled with a fluorophore (fluorescein) was located in the center of the linker; ssDNA was hybridized with two ssDNAs, partially complementary to the aptamers. These DNAs contained 5′- and 3′-fluorescence quenchers. Therefore, only with the simultaneous addition of targets was there a generation of a fluorescent signal—the output signal of the logic. The logic OR gate was organized in a similar way; however, in this case, the fluorescence quencher and fluorophore were located at the 5′- and 3′-ends of short DNA complementary to ssDNA with aptamers. Therefore, the introduction of adenosine and thrombin into the system separately and together led to the dissociation of the quencher and the fluorophore from each other and the restoration of the system output signal. The logic implementation time was 30 min.

The authors proposed a logic scheme for the definition of two types of prions: PrP^c^ and PrP^rev^ [39]. For this, they use two aptamers for different parts of proteins. One of the aptamers is bound to the silicon-coated magnetic microparticles MMPs-Apt1 via a silane spacer and glutaraldehyde. This allows the bound protein to be separated from the solution using a magnetic separator. Furthermore, quantum dots Apt2-QDs functionalized by the second aptamer are added to the proteins bound to the magnetic particles. The aptamer interacts with the protein, and the total MMPs-Apt1–PrP–Apt2-QDs complex is separated from the solution or homogenate using a magnetic separator. The final signal of the logic circuit is the fluorescence of quantum dots which indicate the presence of a protein in the system. To determine the type of prion, guanidinium chloride is used, which, at a certain concentration, denatures the protein. In this case, PrPc is sensitive to the denaturing agent and destroys, ceasing to bind to aptamers. PrPrev is partially resistant to such denaturation and, conversely, begins to bind better to the Apt2-QDs aptamer, since the binding site becomes more accessible in this case. The authors of the study wrote about the implementation of logic gate XOR, in which the input data were PrPc and guanidinium chloride, and OR, in which the input data were PrPrev and guanidinium chloride. The system contains magnetic microparticles modified by one aptamer and quantum dots modified by the second aptamer. The output signal is the fluorescence of quantum dots. In the case of the OR gate, the signal taken as 1 appears both in the presence of one PrPrev and in the joint presence of a prion and a denaturing agent. In the case of the XOR gate, the output signal 1 corresponds only to the presence of PrPc. However, these logic gates cannot be considered complete from the point of view of a standard logic gate, since the introduction of only one guanidinium chloride does not affect the signal of the system in any way. Thus, in the logic OR gate, the signal of guanidinium chloride should be 1, and in this study, it was equal to 0. The same is for the XOR gate. The logic implementation time was 100 min.

Zhang and colleagues implemented the AND logic gate in [79]. The logic was based on the use of fluorescent silver nanoclusters linked to DNA. The use of metal nanoclusters as fluorophores in the creation of biosensors is a relatively new direction [83,84]. Effective quenching of such a fluorophore was found to be possible through photoinduced electron transfer (PET), which was used to create the sensor and logic. Electronic transfer occurred between a peroxidase-like DNAzyme with hemin serving as an acceptor and a silver nanocluster bound to DNA. The DNA structure used in the work was a hairpin and consisted of three parts: an anti-PDGF-BB aptamer, a DNAzyme, and a DNA region associated with a nanocluster. The hairpin loop contained a portion of unassembled DNAzyme and 12 cytosines to stabilize silver nanoparticles. The aptamer and another part of DNAzyme were in the blocked state within the duplex. When a target was added to the system, the aptamer bound to it, and DNAzyme was released from the duplex and collected in an active form in the presence of K^+^ and hemin. Thus, the cytokine PDGF-BB and hemin served as input data. As a result, a decrease in the fluorescence emission of the silver cluster was observed due to the PET between it and the Fe^3+^ core of the hemin. The logic was implemented at a cytokine concentration of 1 nM and hemin of 1 μM; a change in the fluorescent signal by more than 20% was taken as 1. The logic implementation time was 120 min.

There is a logic AND gate, obtained using hollow microparticles, the shell of which consists of layer-by-layer complementary and cross-complementary ssDNA [82]. As ssDNA, aptamers on ATP and vascular endothelial growth factor (VEGF) are involved in the shell construction. Inside the microparticles are Texas Red fluorescent dyes (microcapsules based on aptamer against ATP) and tetramethylrhodamine (microcapsules based on aptamer against VEGF), modified with dextran. The introduction of targets into the system leads to the binding of aptamers to the targets and the destruction of the shell of the microparticles, a result of which the dyes are released into the solution. When implementing the AND logic gate, a mixture of microparticles was used, the input data were ATP at a concentration of 2.5 × 10^−3^ M and VEGF at a concentration of 250 × 10^−9^ M, and the output signal was a fluorescent signal from dyes. The logic implementation time was 50 min. This system has also been used for the controlled release from microparticles of an anticancer compound—dextran-modified doxorubicin. Since cancer cells intensively produce ATP [85] and VEGF [86] a targeted drug release was thus carried out.

### 2.2. The Colorimetric Output in the Solution Due to the Catalytic Labels

This section discusses a cascade scheme with DNA migration and strand displacement during the interaction of aptamers with cocaine and adenosine 5′-monophosphate (AMP) and the release of a catalytically active DNAzyme [58]; it is proposed to use a bifunctional aptamer containing aptamers against AMP and cocaine and blocked by a complementary DNA sequence with DNAzyme [65].

**Figure 2:**
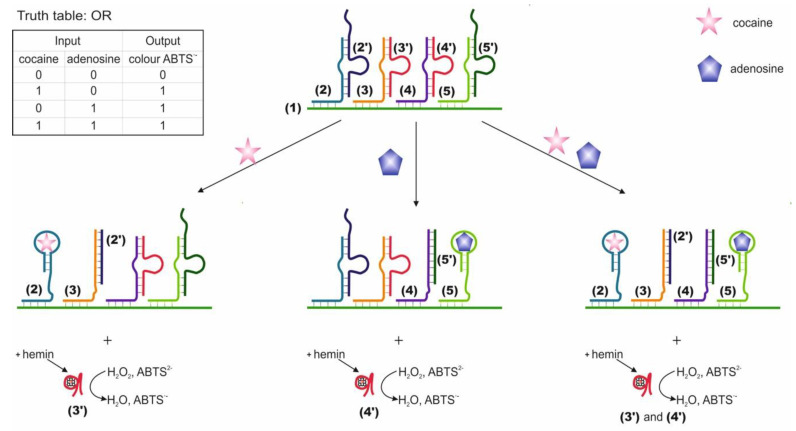
Logic gate OR. ABTS = (2,2-azino-bis (3-ethylbenzothiazoline-6-sulfonic acid) disodium salt). Adapted from Reference [58]. Copyright © 2020, American Chemical Society.

Logic gates OR, AND and XOR based on four DNAs located next to one another and partially hybridized on a common DNA substrate were compiled [58]. The two external DNAs are responsible for target recognition (2 and 5 on the Figure 2) and contain the aptamer sequence, while they are blocked by complementary mediator sequences, 2′ and 5′, correspondingly. Two internal DNAs (3 and 4) are blocked by sequences (3′ and 4′) containing an as-yet inactive DNAzyme catalyzing a peroxidase-like reaction. Mediator sequences (2′ and 5′ in Figure 2) are complimentary to internal DNAs 3 and 4. When a target appears in the system, the corresponding aptamer forms a complex with it. This results in the translocation of the blocking mediator DNA to the adjacent internal DNA with a blocked complementary DNAzyme sequence. During the strand displacement, which starts with the base pairing of the mediator sequences 2′, 5′ and internal DNAs 3 and 4 in the loop region of the duplexes (3/3′ and 4/4′ in Figure 2), DNAzymes 3′ and 4′ are released and duplexes of translocated mediator DNAs and internal DNAs are formed. The liberated DNAzyme forms a complex with hemin and catalyzes the colorimetric oxidation reaction of ABTS (2,2-azino-bis(3-ethylbenzothiazoline-6-sulfonic acid) disodium salt), which serves as the output signal of the system. Targets were used as input data: cocaine and adenosine monophosphate. Accordingly, each external DNA contained an aptamer for one of the targets. The OR logic is implemented when each target results in the release of active DNAzyme (signal timing). The AND logic is formed when each of the targets leads to the release of a part of the DNAzyme; therefore, the pushing DNAzyme is formed only in the presence of both targets. The XOR logic is implemented when each target releases an active DNAzyme, but when released together, the DNAzymes hybridize with each other and become inactive. The incubation time with the targets was 20 min; the time for monitoring the DNAzyme reaction was 2 min. In addition to aptamers, the use of DNA has also been shown as input. In one case, the use of ssDNA as input resulted in the release of the aptamer for thrombin and blocking of its protease activity. In general, the logic system is constructed using a modular design, which allows it to be adapted to various input and output sequences. In this case, the translation of information from one DNA to another is possible. The advantage of the system is that the output response does not go directly but is the result of a DNA cascade: the product of a higher level is activated by a lower one. The disadvantages are that the designed valves are disposable. Also, to implement the logic, high concentrations of targets were used, 1.25 × 10^−4^ M each, and a high ionic strength of solutions (400 mM NaCl). When using the data in the article, it is also worth paying attention to the correctness of the indicated sequences, since sequences 4 and 5, according to those indicated in the article, are hybridized to one fragment of the substrate, which is most likely an error.

**Figure 3:**
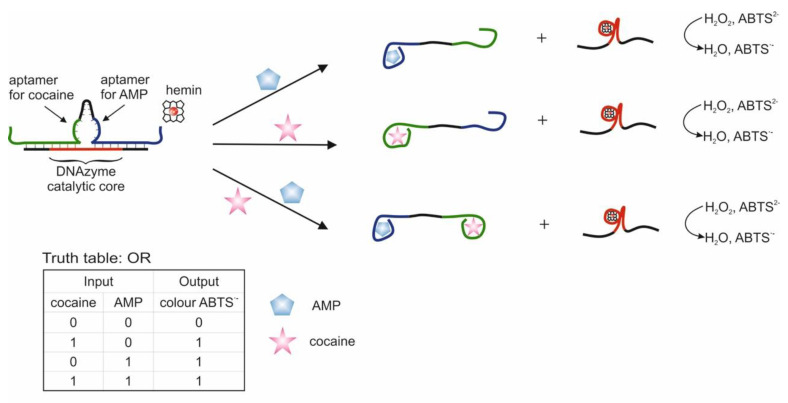
Logic gate OR [65]. AMP = adenosine 5′-monophosphate; ABTS = 2,2-azino-bis(3-ethylbenzothiazoline-6-sulfonic acid) disodium salt.

The use of two aptamers in one DNA sequence has been shown in [65]. This sequence is referred to as a bifunctional aptamer that contains the aptamer to cocaine at one end [50], and at the other end the aptamer to AMP [48]. Targets were used as inputs to the OR logic: cocaine and AMP. The essence of the approach was that the bifunctional aptamer was hybridized with ssDNA to form two duplex fragments and was in a blocked state (Figure 3). The formation of an incomplete duplex with the blocking DNA is due to the fact that in the case of interaction of the aptamer with one of the targets, dehybridization becomes energetically more preferable than in the case of complete hybridization of the sequences. The design of complementary regions is very important, because due to the energy balance between hybridization and dehybridization, the logic of the system is carried out directly: the output signal is 0 and 1. As a result, the blocked bifunctional aptamer dissociated from the blocking DNA in the presence of one or two targets simultaneously. In this case, the colorimetric peroxidase-like reaction of DNAzyme served as the output signal. The input sensitivity in this system was 5 × 10^−6^ M for cocaine and 1 × 10^−5^ M for AMP. The system output signal was formed within 62 min: 60 min of incubation with targets and 2 min of colorimetric measurement. In the second case, the separation of aptamers from the blocking DNA was detected by impedance spectroscopy, and the change in the resistance of interfacial electron transfer on the electrode surface served as the output signal. In this case, the blocking DNA was immobilized on gold electrodes. The sensitivity of this method of generating the output signal was the same as the previous one. In the third case, the blocking sequence was immobilized on the surface of an ion-sensitive field-effect transistor. Dehybridization of the aptamer with the target from the surface led to a change in the potential of the second source, which was reflected in the change in the potential of the second source. The output signal of the logic in this case was the gate-source potential, which assumed a constant value after 15 min. On the one hand, the use of bifunctional aptamers is an interesting solution, especially when introducing logical operations into sensing, when several markers are important for diagnosis simultaneously: their joint presence or the presence of at least one. Also, many known aptamers in combination with DNAzymes will make it possible to create various logical operations, including cascades. At the same time, bound aptamers can impact on each other (conformationally or partially complementary), as a result of which the efficiency of their interaction with targets can deteriorate. In addition, at this stage, the approach does not accurately identify the connection that triggers the response.

### 2.3. Logical Gates Based on Gold Nanoparticles

Gold nanoparticles (AuNPs) are used in the construction of aptamer-based logical operations due to the fact of their unique properties: surface plasmon resonance (SPR) [87], which is dependent on size and the distance between the particles, high molar extinction coefficient and highly efficient quenching of various fluorophores [74]. Taken together, these properties lead to a possibility of a fast, sensitive, and visual on-the-spot analysis.

Existing AuNP-based logic gates can be divided into two groups according to the principle of output signal formation: colorimetric [38,41,54,55,66,67,68,69,71,72] and fluorescent [38,57,74,75].

Changes in colorimetric output signals occur due to the fact of changes in the optical density of the AuNP-containing solution, caused by aggregation or disaggregation [66,72] of AuNPs in the process of target recognition by aptamer. This effect is based on surface plasmon resonance. For example, the most common average nanoparticle size is 13 nm. Aggregation of such AuNPs leads to a shift in surface plasmon resonance from λ_max_ ~ 525 nm to 560 nm. Visually it can be described as solution changing color from red (AuNP not aggregated) to purple (AuNP aggregated).

The fluorescent output signal of logic gates is based on fluorescent label quenching (Cy5, TAMRA, FITC, etc.) in the vicinity of AuNP [38,57,74,75]. When the fluorescent label is removed from the nanoparticle’s surface, fluorescence is restored. The label is distanced when the labeled aptamer interacts with the target. The principle of AuNP fluorescence quenching is based on fluorescence resonance energy transfer (FRET) or inner filter effect (IFE).

It is worth noting the possibility of organizing logic gates with multi-inputs, which, in addition to targets, also use DNA as input data [57,66,69].

The AuNP-based schemes can be divided into schemes using covalent modified or unmodified nanoparticles.

The former is more commonly used and is more universal; however, it is time-consuming and requires complicated modification procedures [88]. In this case, nanoparticles are covalently modified by aptamers, and logic inputs are usually aptamer targets. The latter case is not as common and is more rare, and it is based on physical sorption of ssDNA (aptamers) onto AuNP surface, which prevents their aggregation by electrostatic repulsion after salt addition. When an aptamer–target complex is forming, it causes formation of a rigid DNA structure which cannot be sorbed onto particle surface [55,71]; also, sorbed aptamers can desorb during target addition [41,42]. As a result, particles aggregation occurs after salt addition. Input data in unmodified particles logic are usually targets and aptamers.

Comparison of strategies using covalent modified [72] и and unmodified [55] AuNPs in OR and AND logic gates, where aptamer targets (cocaine and adenosine) were used as inputs, has shown that detection limits of these compounds were practically the same. Precisely, in the case of unmodified AuNP, detection limits for OR logic gate were 0.2 mM for adenosine and 0.2 mM for cocaine; for the AND logic gate—0.4 mM for adenosine and 0.6 mM for cocaine. In the case of modified AuNPs, for the OR logic gate, a discernible signal was obtained for 0.1 mM adenosine and 0.1 mM cocaine; for the AND logic gate, 0.2 mM/0.5 mM for adenosine and 0.5/0.2 mM for cocaine. K_d_ for the cocaine aptamer is 5 µM [49,50], and K_d_ for the adenosine aptamer is 6 ± 3 µM [47].

A separate direction of research is the development of lateral flow strip biosensors [54,68,69] and logic gates based on them. In this case, modified AuNPs cause the generation of output signals, which is the coloration of test areas, visible by the naked eye. This approach is fast and simple at the starting point of a logic operation.

#### 2.3.1. Logic Gates Based on Covalently Modified Gold Nanoparticles

This section describes the use of DNA nanomachines with the participation of a nicking enzyme and polymerase to amplify a signal from a target, while the output signal is the aggregation of modified ssDNA gold nanoparticles [66]. It shows a study at the level of one gold nanoparticle modified with ssDNA (aptamer) with a fluorescent label [75]; the development of logic gates based on disaggregation of nanoparticles with switching of aptamers from dsDNA to the target [72]; a similar system which implies reading the output signal from two channels: fluorescent and colorimetric, the first based on quenching of the fluorescent label by AuNPs and the second on the aggregation of nanoparticles [38]. A combination of switching an aptamer from dsDNA to a target with a chain replacement strategy is known; the system used three-input data [57]. Also a system based on a combination of a fluorescent labeled aptamer, which is immobilized on the AuNPs surface in the form of a hairpin, and different metal ions is obtained recently [89].

There is the work which describes a multi-component logic system based on using multiple (more than two) inputs to produce an output signal [66]. The output is a colorimetric signal from logically controlled aggregation of AuNP caused by hybridization of ssDNA bound to nanoparticles and ssDNA produced as an output signal of a DNA nanomachine. This DNA nanomachine is a system consisting of DNA polymerase with strand displacement activity (Klenow fragment), dNTPs mix, a nicking enzyme that cuts one strand of a DNA duplex at a specific sequence (nicking site), and a template DNA composed of three parts: a 3′-part (an anti-analyte sequence such as an aptamer), a middle part (an anti-nicking site), and a 5′-part (an anti-output sequence). The mechanism of signal conversion is as follows: (1) An analyte (target or ssDNA) binds to the 3′-part to make the 3′-terminus bind to a 5′-region of its own template. (2) The 3′ terminus of the template is extended by DNA polymerase with dNTPs. (3) The resultant DNA duplex is nicked at the nicking site to produce an output single-stranded DNA (ssDNA). (4) The 3′-terminus at the nicking site is extended again due to the strand displacement activity of DNA polymerase. (5) As a result of the cycle, a large amount of ssDNA is produced. Inputs of DNA nanomachine, and, by extension, logic inputs are ssDNA and aptamer targets (Hg^2+^ ions). Foundational AND logic gates with three inputs (A AND B AND C) were constructed using heterogeneous DNA nanomachines, which convert each input into corresponding ssDNA (A → a, B → b, C → c). Strand *b* was able to bind strands *a* and *c*, forming an *abc* complex by hybridizing with 3′- and 5′-terminuses, respectively. Nonhybridized fragments of *a* and *c* strands of the *abc* complex can form duplexes with *x* and *y* strands bound to AuNPs, leading to AuNPs aggregation. Logic output is the colorimetric signal. Changing DNA-nanomachine type from heterogeneous to partially homogeneous (A → a, B → a, C → c) or fully homogeneous (A → a, B → a, C → a) enabled construction of integrative AND/OR ((A OR B) AND C) and OR (A OR B OR C) logic gates, respectively. In addition to that, a logic gate (A AND (NOT B) AND C) containing a NOT operation was constructed. The Hg^2+^ ion binding sequence was used as an aptamer. The logic processing time was approximately 3 h.

Another publication describes XOR logic based on AuNPs in homogeneous solution and on a single-nanoparticle level using total internal reflection fluorescence (TIRF) microscopy [75]. This system consisted of 13 nm AuNPs, which were functionalized by ssDNAs with Cy5 labels. AuNPs quench fluorescence on a certain distance from fluorophore due to the FRET [74]. One AuNP was modified with ssDNA in a hairpin shape and adenosine aptamer, which contained a complimentary sequence on the terminus removed from the nanoparticle’s surface. Thus, ssDNA fluorescence was quenched from the start, and the aptamer label fluorescence was not quenched [90,91]. In addition to that, nanoparticles were covered by “helper” DNA (T_10_) for stabilization. Adenosine and complimentary ssDNA were used as input data. Adenosine addition led to structural changes in the aptamer, and Cy5 label came close to a nanoparticle surface, triggering fluorescence quenching. Addition of ssDNA complimentary to hairpin ssDNA caused straightening of the hairpin and removal of Cy5 label from the surface, thus restoring its fluorescence. The fluorescent output signal was considered “1” if the signal change was more than 5% from the base value and “0” if the change was less than 5%. As dissociation constants for aptamer (6 µM [47]) and complimentary DNA (nM range [92]) were considerably different, optimal concentration values were needed to be found for XOR logic construction: each target gives a “1” signal, together they give a “0” signal. The molar ratio of adenosine/complimentary DNA was 5000:1 (250 µM versus 50 nM). It should be noted that introduction of a sequence complimentary to aptamer provided better target binding, as the binding domain was removed from the nanoparticle’s surface in this case [93].

An example of AND and OR logic gates construction is a system using modified AuNPs [72]. Adenosine [47] and cocaine [49] aptamers were used. As with the previous cases, logic was based on aggregation/disaggregation of nanoparticles causing the change in color of the solution. Initially, two AND logic gates were constructed so that only when the two targets (adenosine and cocaine) were added, disaggregation occurred, and the signal exceeded the threshold for “1”. In the first version of AND logic, two groups of nanoparticles were used: one group was modified with identical probes, the other with various probes. The particles were bound to each other due to the hybridization of their probes with two linkers: one containing adenosine aptamer, the other a cocaine aptamer. In the case of addition of both targets, they formed a complex with aptamers and nanoparticles were separated from each other; as in this case, the number of complimentary bases was reduced to five, which is not enough to maintain hybridization at room temperature. In the second version of AND logic, three groups of nanoparticles were used, each with its own type of probe. They were also bound through two linkers with adenosine and cocaine aptamers. In this case, the presence of a single target lead to a partial disaggregation and only simultaneous addition of two targets produced a signal above threshold value. The OR logic gate was constructed using two types of nanoparticles, each with its own type of probe. Nanoparticles were bound to each other through common linker containing both aptamers. The addition of both targets and each target individually led to nanoparticle disaggregation. The logic processing time was less than 5 min. Aggregation/disaggregation of nanoparticles was detected using UV-Vis spectrophotometry on 520 nm and 700 nm wavelengths. Aside from adenosine and cocaine aptamers, a logic using K+ aptamer was constructed [94]. In the presence of K^+^ ions the aptamer formed G-quadruplexes, and gold nanoparticles were separated from each other. The AND logic gates were based on combinations of input data: adenosine and K^+^, cocaine and K^+^.

**Figure 4:**
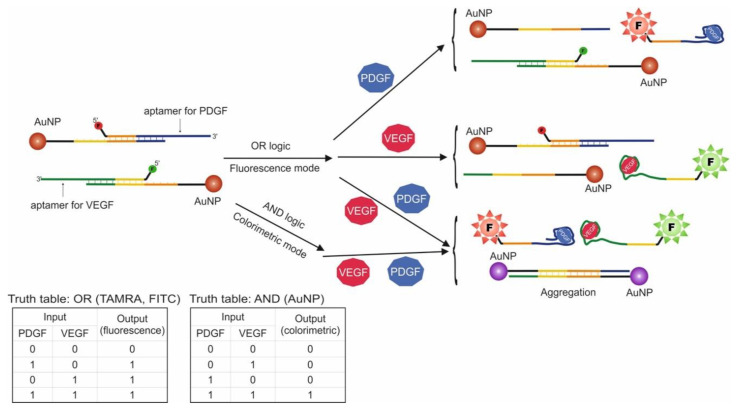
OR and AND logic gates. PDGF = platelet-derived growth factor, VEGF = vascular endothelial growth factor, F—fluorescent label. Adapted from Reference [38]. Copyright © 2020, American Chemical Society.

Another logic construction using AuNPs is known. In this work, aptamers for VEGF and PDGF were used, and these proteins served as input data (Figure 4) [38]. These proteins are biomarkers for cancer cells, and the goal of logic construction was cancer screening and further treatment. The logic system was designed to signal the presence of one or both targets (OR logic gate) using fluorescence and the presence of two targets simultaneously (AND logic gate) using a visual change in the solution color from red to purple due to the properties of AuNPs. To this end, AuNPs with two types of attached oligonucleotide probes were used: one type was hybridized with VEGF aptamer, the other with PDGF aptamer. Additionally, VEGF aptamer contained a FITC label, and the PDGF aptamer contained a TAMRA label. It should be noted that AuNP nanoparticles quench label fluorescence at a close distance, which is explained by the different energy transfer mechanisms. As a result, addition of input data into the system leads to a fluorescent signal dependent on the added target (OR logic), as aptamer dehybridizes from the probe and nanoparticle and binds to the protein. At the same time, the two types of nanoparticle probes have fragments complimentary to each other. As a result, in the presence of both proteins, the probes are detached from aptamers and are hybridized with each other, causing aggregation of nanoparticles and shift in surface plasmon resonance from λ_max_ = 525 to 560 nm. Visually this can be described as the solution changing color from red (unaggregated AuNPs) to purple (aggregated AuNPs). In this way, AND logic and OR logic in the presence of both targets (existence of fluorescent signals) are realized. The developed system can simultaneously function in AND/OR logic, depending on the chosen detection method. With the use of AND logic, the simultaneous presence of PDGF and VEGF in concentrations of 1 nM each were detected. The extent to which AuNPs are aggregated is dependent on the target concentration, as each nanoparticle contains more than one oligonucleotide probe. As a result, the more probes are detached from aptamers, the more 3D AuNP aggregation occurs. The logic processing time was 120 min.

Another example of a three-input AND logic gate is the following [57]. In this study, the authors used an ATP aptamer. The described method was based on AuNPs modification by ssDNA, which was then partially hybridized with an ATP aptamer and two ssDNAs, one of which contained a fluorescent label (FAM). In its original state, the label was close to the nanoparticle’s surface and therefore quenched. The ATP and two ssDNA, which were complimentary to the two hybridized ssDNA, were used as input data. A fluorescent signal corresponding to a “1” output was generated only when all three inputs were present. In this case, a cascade mechanism could be observed: separation of aptamer from AuNP-based sequence and consecutive separation of the first and second hybridized DNA. In this process, two main mechanisms were at work: aptamer switching from dsDNA to target–aptamer complex when the target was added and toehold-mediated strand displacement for ssDNA as input data. The logic processing time was more than 17 min.

System, consisting of AuNPs conjugated with Cy3-tagged aptamer against chloramphenicol, was used to fabricate YES, PASS 0, INH, NOT, PASS 1, and NAND logic gates [89]. Initially ssDNA with the aptamer was in the form of a hairpin so the Cy3 label situated in the close proximity to the AuNPs surface. According to the FRET, Cy3 as donor and AuNPs as recipient, the fluorescence of the label was quenched. Addition of chloramphenicol caused switching of the aptamer from dsDNA (hairpin) to a complex with the target and subsequent restoration of the fluorescence due to removal of the Cy3 label from the gold surface. Also, it has been well documented that various metal ions are able to interact with DNA at different extent [95,96]. Using the principle of a metal ion–mediated fluorescence switch together with a strong metal ion chelator, the fluorescence could be modulated by adding metal ions and EDTA sequentially. Chloramphenicol and Ni^2+^ (quenched the fluorescence of modified AuNPs even in the presence of the target) served as chemical inputs for YES, PASS 0, and INHBIT logic gates where modified AuNPs was the system under the consideration. Ions of Hg^2+^ (enhanced the fluorescence of modified AuNPs in the presence of the target) and Ni^2+^ were used as inputs for PASS 1 and NOT gates while aptamer-modified AuNPs and chloramphenicol served as the system under consideration. For the system consisting of modified AuNPs, chloramphenicol and Hg^2+^, NOT logic gate was obtained using ions of Ni^2+^ as input. Vice versa, for the system consisting of modified AuNPs, chloramphenicol and Ni^2+^, PASS 0 logic gate was obtained using ions of Hg^2+^ as input. Logic gates PASS 1 (two variants) and NAND were fabricated on the basis of modified AuNPs, chloramphenicol and EDTA as the initial system and ions of Hg^2+^ and Ni^2+^ as inputs. The fluorescence intensity served as the output signal for all gates: high intensity-1, low intensity-0. The logic processing time was 60 min (50 °C).

##### Lateral Flow Strip Biosensors

A special place among biosensors belongs to lateral flow strip biosensors (LFSBs), which are easy to use, provide a quick response, reliable, and low cost. Intensive research has focused on the development of new types of LFSBs, and some of the more recent examples are reviewed in [97]. The basic principles of constructing logic gates based on aptamers and LFSBs are the use of AuNPs (with immobilized DNA) due to the fact of which staining of the test zones occurs, as well as for the implementation of various gates, aptamers could use as they are or divided into parts.

This section discusses test strips based on split aptamers, one of the split parts contained AuNP [54]; also shown is the fabrication of logic gates based on AuNPs modified with two different ssDNA in the form of hairpins with biotin at the free end [68]; based on the previous scheme, eight combinations of various gates were created, where, in addition to targets, the aptamers themselves were used as inputs [69].

**Figure 5:**
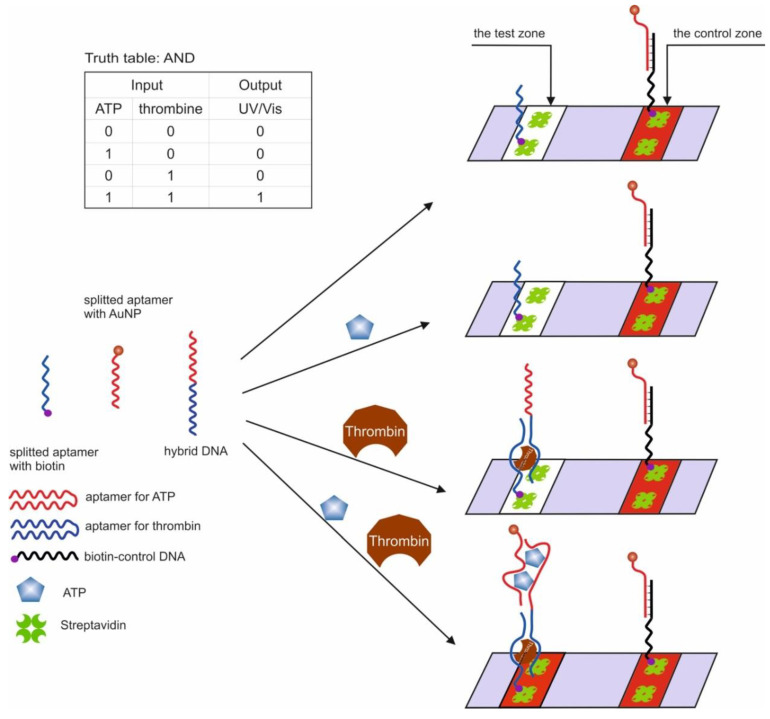
The AND logic gate. ATP = adenosine triphosphate. Adapted from Reference [54]. Copyright © 2020, American Chemical Society.

The first example of the use of aptamers in the construction of logic OR and AND gates in test strips was the work in [54]. The authors used aptamers for thrombin (15 bp) [44] and ATP (27 bp) [47,51], split into two parts [56,98] and interconnected so that two hybrid sequences were obtained. It has previously been shown that cleaved aptamers are able to retain their target affinity [99,100]. When a target was introduced into a system consisting of such hybrid sequences, a three-component complex was formed, induced by self-assembly of cleaved fragments of the aptamer. The OR gate was constructed as follows: one of the hybrid sequences contained biotin at one end and the second hybrid sequence contained gold nanoparticles (AuNP), which were responsible for the colorimetric output signal. The test strip, in turn, had a sample zone with streptavidin and a control zone with ssDNA, partly complementary to the AuNP sequence. When a target was introduced into the system, it bound to both hybrid DNAs, one of which held the entire structure in the streptavidin zone, which can be observed with the naked eye. The logic AND gate was realized using three fragments of cleaved aptamers: a hybrid sequence, a fragment of an aptamer for thrombin with biotin, and a fragment of an aptamer for ATP with AuNPs (Figure 5). The staining of the sample area occurred only in the presence of both targets in the system. The authors note that the duration of the logic implementation did not exceed 45 min. The proposed logical operations are simple to execute, economical, and require neither technical knowledge nor expensive complex tools. At the same time, the application of this approach to other targets remains questionable, since it is not known whether it is possible to separate aptamers in this way while maintaining their affinities. 

**Figure 6:**
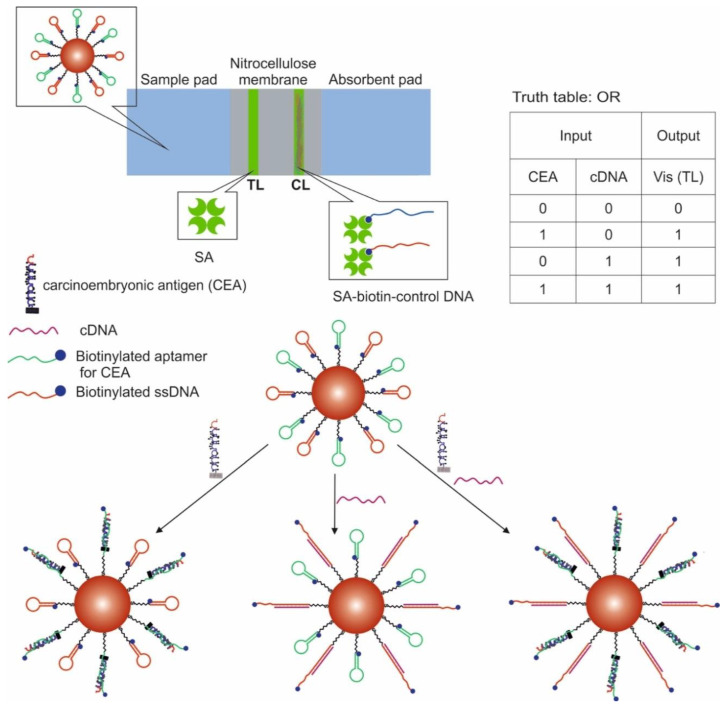
The OR logic gate. Adapted from Reference [68]. Copyright © 2020 Elsevier B.V.

Another work shows the creation of logic OR and INHIBIT gates using LFSBs and AuNPs [68]. AuNPs were modified with a thiol-containing aptamer for carcinoembryonic antigen and ssDNA. At the same time, both the aptamer and ssDNA were in the hairpin state, the non-immobilized end of which contained blocked biotin. In the presence of a target for the aptamer and complementary DNA for ssDNA, the hairpins opened, and biotin could interact with streptavidin. When implementing the OR logic (Figure 6), carcinoembryonic antigen (200 ng/mL, discernible by visual detection) and complementary DNA (1 μM, discernible by visual detection) were used as input data; one AuNP was modified with an aptamer and ssDNA. The test zone containing immobilized streptavidin was stained in the presence of the input data due to the retention of modified gold nanoparticles. The control zone contained streptavidin-labeled DNA complementary to the aptamer and ssDNA and retaining gold nanoparticles. When implementing the logic gate INHIBIT, the carcinoembryonic antigen (200 ng/mL) and free aptamer (1 μM) in the form of a hairpin were introduced into this target as input data; AuNPs were modified only with the aptamer. As a result, the test area was stained only in the presence of carcinoembryonic antigen. In the presence of both inputs, the free aptamer competitively formed a complex with the target, so that the aptamer bound to the nanoparticles did not interact with the antigen in a sufficient amount and biotin was not released. Output signal 1 corresponded to the stained, and output signal 0 corresponded when there was no staining. The incubation time of AuNPs with the input data was 30 min, and the logic implementation time was 20 min.

This was a continuation of the previous work [69]. On the basis of four elementary logic gates OR, AND, INHIBIT, and NAND, eight different combinations were composed: AND–OR; AND–INH; OR–INH; INH–NAND; AND–OR–INH; AND–INH–NAND; OR–INH–NAND; AND–OR–INH–NAND. The logical schemes were based on the use of AuNPs modified by aptamers, which provided visual staining of the test zones due to the fact that they were retained in them. Proteins were used as targets for aptamers: thrombin, mucin 1, and carcinoembryonic antigen. The authors also used in their work the fact that the same aptamer can interact with different parts of the protein, with a sandwich mechanism being implemented: two aptamers and one target. The input data for various logic gates were aptamer targets, modified nanoparticles, and aptamers. AuNPs of one species were modified according to the following scheme: one biotin-containing aptamer in the form of a hairpin per protein; aptamer to another protein. Test zone 1 (TZ1) contained immobilized streptavidin which captured the biotin-containing aptamer when the hairpin opened in the presence of target 1. As a result, in the presence of target 1, this zone was colored red due to the AuNPs. Test zone 2 (TZ2) contained immobilized streptavidin bound to the aptamer on another target 2. In the presence of target 2, this zone also turned red, since a sandwich complex was formed between the aptamer with nanoparticles, target 2, and the aptamer with streptavidin. The control zone (CZ) contained immobilized streptavidin modified with DNA complementary to one of the aptamers on the nanoparticles. Thus, AuNPs were retained and stained in the CZ. Output 1 was when the test area was stained and output 0 was when no staining occurred. The holding of targets with nanoparticles before being applied to the test strip lasted for 12 h. The movement of nanoparticles in the test strip was provided by capillary forces. The use of combinations of logic gates implied the introduction of more than two targets into the system. In this case, the order of the introduction of the targets was important, and the permutations led to the generation of various signals of the test system. In bio-computing, this fact can be used to generate passwords for opening a lock (keypad–lock system). The detection limits for thrombin, mucin 1, and carcinoembryonic antigen were 1.61 nM (linear range 3.2–250 nM), 1.13 nM (linear range 1.6–400 nM) and 0.7 nM (linear range 0.8–300 nM), respectively. It is worth noting a certain ambiguity in the article due to the fact that the indicated sequences of aptamers for one target are identical: a biotin-containing aptamer and an unlabeled aptamer. At the same time, the biotin-containing aptamer was presented in the form of a hairpin, while the second was described as an unfolded ssDNA.

##### Mesoporous Silicon

The AND and OR logic gates were created using modified AuNPs and modified mesoporous silicon particles [101]. Mesoporous silicon contained covalently bound ssDNA [102] which hybridized with ssDNA bound to AuNP. Thus, the silicon pore was physically sealed with a nanoparticle, while the pore cavity was preliminarily filled by diffusion with the dye rhodamine B. The release of rhodamine B caused the output luminescent (λ = 570 nm) logic signal. The logic OR gate was implemented with the inclusion of an aptamer for K^+^ in the ssDNA covalently bound to AuNPs. The input signals of the logic gate were K^+^ and temperature rise (namely temperature rise). As a result of the introduction of K^+^ ions into the system, the aptamer bound to them, and ssDNA with AuNP dissociated from the ssDNA of mesoporous silicon (duplex length 17 bp). This led to the release of the luminescent dye from the pore. In the same way, the DNA duplex was destroyed with increasing temperature. The logic AND gate implied the inclusion of aptamer for adenosine in ssDNA nanoparticles, and aptamer for cocaine in ssDNA silicon. Only the joint addition of targets promoted the dissociation of the duplex (duplex length 22 np) and the appearance of the output signal. The implementation of the logic was observed for 70 min. The possibility of introducing mesoporous silicon particles filled with an anticancer compound, camptothecin, into cancer cells and the implementation of the AND logic using the example of cocaine and adenosine targets was shown. Cell survival decreased by 32–38% in this case.

#### 2.3.2. Logic Gates Based on Unmodified Gold Nanoparticles

It is known that ssDNA can be sorbed on the surface of AuNPs, in particular, aptamers [103], while dsDNA [104] and the aptamer–target complex is not retained on the surface of AuNPs. According to modern studies, this is due to the hydrophobic effects [103], the rate of DNA adsorption depends on the nucleotide composition [105] and on the salt composition of the solution. This property is often used when organizing logic operations based on unmodified nanoparticles. There are gates in which the complex of the aptamer with the target is not sorbed on AuNPs (premix of the aptamer with the target and subsequent addition to the AuNPs) [55,71], as well as gates in which the aptamer is desorbed from the surface of nanoparticles in the presence of a target [41,42]. The latter indicates that the affinity of these aptamers for targets is higher than sorption on nanoparticles. When a salt solution is added at a high concentration, unmodified AuNPs aggregate, while the nanoparticles coated with ssDNA remain in a dispersed state, which is probably due to the electrostatic repulsion. Thus, the main output signal of the logic gates is the colorimetric signal from the aggregation of nanoparticles. This differs from gates based on covalently modified AuNPs. The main input data are targets for aptamers and the aptamers.

The strategy of using unmodified AuNPs in logic gates involves the use of practical schemes, which more often use specific targets for the real analysis. Thus, the recognition system for bisphenol A and bisphenol S [42] and d/l enantiomers of arginine [41]. Also, when implementing the logic, the unique properties of the very system of consideration are involved: the INHIBIT logic gate with the target arginine vasopressin, which causes the aggregation of AuNPs [71].

The inclusion of AuNPs [106] and other NPs into the 3D hydrogel network [107], which determines the color of the gel and the solution above it, can be considered a special type of construction of logic gates.

In addition to logic gates, there are logic circuits. For example, they use AuNPs with adsorbed aptamers, while the color of the sample when salt is added depends on the type of aptamer and the introduced protein target. It can be used to discriminate proteins [108]. There are also sensors based on covalently unmodified AuNPs and aptamers [109,110,111].

This section discusses the use of split aptamers [55]; the use of the same aptamer to bisphenol A and bisphenol S is shown [42]; a logic gate for the determination of arginine vasopressin is presentened [71]; a gate for the detection of D-arginine and L-arginine is shown [41].

**Figure 7:**
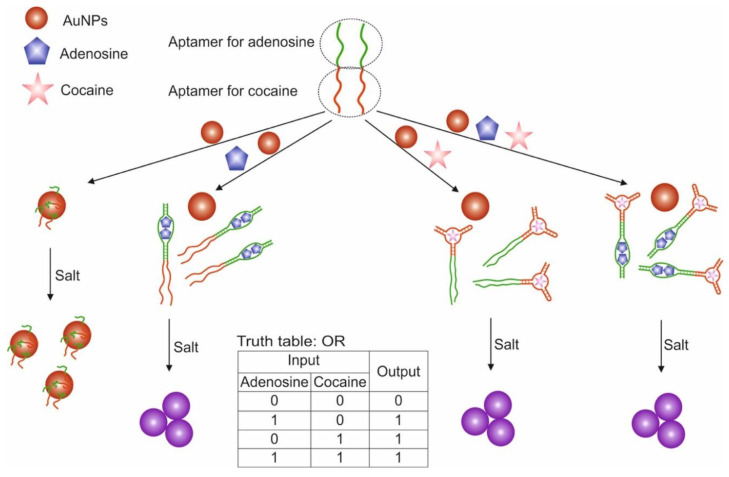
The OR logic gate. Adapted from Reference [55] by permission of The Royal Society of Chemistry.

A variant of the OR and AND gates built based on unmodified AuNP and split aptamers is presented in [55]. The study used aptamers for adenosine and cocaine, which were previously shown to retain their functionality when cleaved into two fragments each [100,112]. To construct the OR logic (Figure 7), we used two integrated DNA sequences (OR-1 and OR-2), one of which contained one fragment of aptamer for adenosine and cocaine in the 3′-5′direction, the other contained the second fragments of aptamers in the direction 5′-3′. The combined DNA retained the ability to recognize targets [98]. AuNPs coated with adsorbed combined DNAs in the absence of targets were in a stabilized non-aggregated state when salt was added due to the electrostatic repulsion of negatively charged DNA strands. The addition of one target or both led to the binding of aptamers to them and the aggregation of AuNPs upon addition of salt: output signal 1 of the OR gate. In this case, the aptamers in the complex with the target were unable to sorb on the surface of nanoparticles and stabilize them, which is probably due to the more rigid structure of the aptamers in the complex as compared to the free state. The output signal of the logic was a colorimetric signal associated with the aggregation of AuNPs: dispersed nanoparticles—red (λ = 520 nm), aggregated—purple (blue) (λ = 650 nm). The AND gate was composed using three DNAs: OR-2 and separately two fragments of aptamers for adenosine and cocaine. Aggregation of nanoparticles above the threshold value occurred only with the simultaneous presence of cocaine and adenosine in the system: output signal 1. The advantage of the proposed logic is that it is simple to implement and does not require modification of nanoparticles. However, questions remain open related to the possibility of using cleaved aptamers for other targets with preservation of their affinity, as well as the use of compounds with a color as targets. The logic time was approximately 10 min. It is worth noting the detection limits of cocaine and adenosine using the proposed logical valves: 0.2 mM for adenosine and 0.2 mM for cocaine for the OR valve; 0.4 mM for adenosine and 0.6 mM for cocaine for AND valve.

The study showed the creation of IMPLY (IMPLY1, IMPLY2, IMPLY1 + IMPLY2) and OR logic gates using AuNPs and bisphenol aptamer [42]. In this work, an anti-bisphenol A aptamer was used [67,113], which, as has been shown, is also capable of recognizing bisphenol S. When implementing the IMPLY1 gate, the input data of the logic were an aptamer against bisphenol A and bisphenol S. The colorimetric signal from solution of AuNPs, corresponding to their aggregation (A_660_/A_520_ > 0.2) corresponded to output 1. At ambient temperature, the aptamer is in a random coil state and can be naturally sorbed on the surface of AuNPs through the van der Waals forces, thus stabilizing AuNP against aggregation induced by the addition of salt (melamine in this work). In this case, the particles remain in a dispersed state, and the solution with them has a red color. The introduction of only bisphenol A into the medium or simultaneously the aptamer and bisphenol A leads to aggregation of nanoparticles and a change in the color of their solution from red (λ = 520 nm) to blue (λ = 660 nm) upon the addition of salt. The results obtained indicate that the complex of the aptamer and the target was not sorbed on the surface of nanoparticles. In a similar way, the logic gate IMPLY2 was compiled, where the aptamer against bisphenol A and bisphenol S were used as input data. The combination of valves IMPLY1 + IMPLY2 implied the presence of three inputs: aptamer for bisphenol A, bisphenol A, bisphenol S; the output signal, taken as 1, met the requirement of nanoparticle aggregation. In this case, AuNPs were in a dispersed state only in the presence of one aptamer in the system (1, 0, 0). The logical OR gate was based on IMPLY1 + IMPLY2, where the input data were bisphenol A and bisphenol S, and the aptamer was initially present in the system at a fixed concentration. As a result, the addition of one target (0.1; 1.0) or both (1.1) led to the aggregation of nanoparticles, which indicated desorption of the aptamer from the surface of nanoparticles and interaction with the targets. Based on the OR gate, the detection limits and linear ranges for bisphenol S and bisphenol A were determined and were 1.3 ng/mL and 2.5–75 ng/mL and 1.5 ng/mL and 4.4–66 ng/mL, respectively. To differentiate between bisphenol A and bisphenol S, solid-phase extraction was used with further introduction of the extracts into the OR gate. The separation was based on the different solubility of bisphenols in solvents. The logic implementation time was 18 min.

The INHIBIT gate was developed based on an aptamer against D-arginine vasopressin and citrate-containing AuNPs [71]. Arginine vasopressin (Cys-Tyr-Phe-Gln-Asn-Cys-Pro-Arg-Gly-NH2) is a cyclic hormonal peptide; two cysteines form a disulfide bridge. It exists in the form of two enantiomers d and l. The presence of a disulfide bridge allows the peptide to be adsorbed on the surface of AuNPs [114], causing their aggregation. The aptamer versus the d-enantiomer [115] interacted with the target and prevented its interaction with nanoparticles and their aggregation. Logic inputs were d-arginine vasopressin and aptamer against it. The output signal is the visually and spectrophotometrically detected aggregation of nanoparticles; as 1, the signal corresponding to the aggregated state is received. The logic implementation time was 40 min.

The logic OR gate based on the arginine aptamer [116] and unmodified AuNPs for the selective determination of chiral enantiomers of arginine is described in [41]. The enantiomers of arginine, d-arginine and l -arginine were used as input data into the logical system, and the aggregation of nanoparticles served as the output signal (in this work, the aggregation was determined by the TEM (Transmission Electron Microscopy) method). In the absence of arginine enantiomers, the fluorescein-containing aptamer was adsorbed on nanoparticles, and the fluorescence of the label was quenched by nanoparticles. The introduction of d/l-arginine into the system led to the formation of a complex of the aptamer with targets and its desorption from the surface of nanoparticles that resulted in the restoration of fluorescence, while the aggregation of nanoparticles occurred to varying degrees. The degree of aggregation depended on the type of target introduced, since the researchers showed that the aptamer had a higher affinity for L-arginine compared to d-arginine. Therefore, the presence of targets at the same concentration led to the formation of large aggregates in the case of L-arginine. The logic action time was 45 min—time of incubation of AuNPs with pre-sorbed aptamer and targets. The calculated limit of detection for L-arginine is 1.9 nM.

##### Hydrogels

The creation of a logic gates based on hydrogels with included aptamers allows one to obtain smart materials that can change their properties depending on the presence of targets in the medium. This allows control of the physical properties of the material, which leads either to the release of the payload enclosed in the polymer network [106] or to a change in the physical size of the hydrogel sheet (compression) [107]. AuNPs and silicon nanoparticles serve as labels, enclosed in the polymer network and reflecting changes when targets are added.

**Figure 8:**
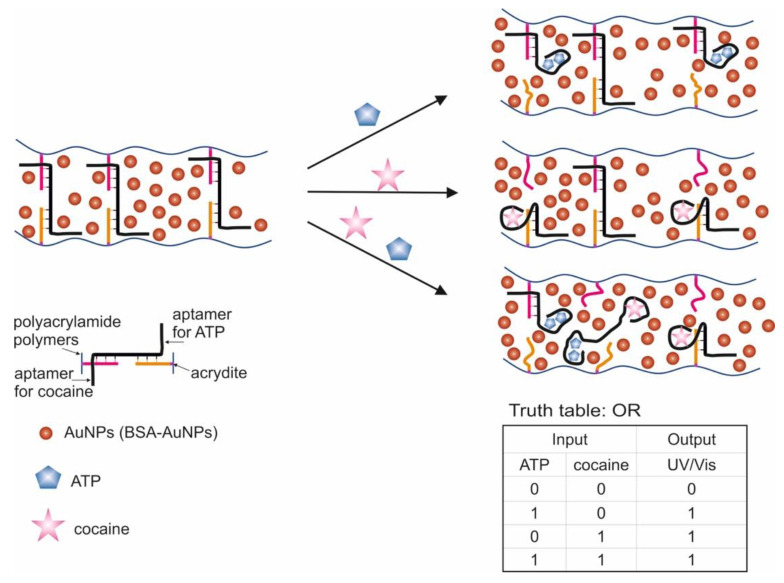
The OR logic gate. ATP = adenosine triphosphate. Adapted from Reference [106] by permission of The Royal Society of Chemistry.

A logic system was built based on hydrogels cross-linked with aptamers [106]. Depending on the type of cross-linking (hybridization) of the aptamers, AND and OR gates were created. The input data were targets: ATP and cocaine. The creation of hydrogels using a cocaine aptamer was known earlier [117]. When creating the AND gate, aptamers on ATP [47] and cocaine [49,50] were used as separate sequences. The OR gate was constructed using a sequence containing both aptamers (bifunctional aptamer). The essence of the method was that two acridite-containing DNA (S1 and S2) were copolymerized with linear polyacrylamide. In the case of the AND gate, one of these DNAs (S1) contained the aptamer on ATP, and the cross-linking DNA (L1) contained the aptamer on cocaine. In this case, the L1 cross-linking DNA hybridized with S1 and S2, and a stable spatial viscous hydrogel was formed as a result of hybridization. Sequences S1, S2 and L1 were hybridized in such a way that a Y-shaped structure was obtained: each of the sequences contained two domains complementary to the other two sequences. AuNPs bound to bovine serum albumin were used to visualize gel formation and disintegration upon addition of targets (input data). AuNPs have a high extinction coefficient, and coloration can be observed colorimetrically and with the naked eye [118]. When ATP and cocaine were added to the gel (AND gate), it disintegrated, and the nanoparticles were released into solution. The introduction of only one target did not cause the destruction of the gel, since the hydrogel units remained connected due to the fact of either the linking sequence or due to the S1. The OR logic gate was designed in a similar way (Figure 8). Only in this case did acridite-containing DNA not contain aptamers, and a bifunctional aptamer formed by cross-linking of aptamers to cocaine and ATP was used as the linking sequence. DNA S3, S4, and L2 formed an H-shaped structure. As a result, the introduction of only one target was sufficient to break the gel and release AuNPs, which led to the color of the solution. The realization time of logic operations was 25 min maximum. The detection limits for the developed system were 20 μM for cocaine and 50 μM for ATP. The logic proposed by the authors allows one to obtain hydrogels based on aptamers with compounds incorporated into the interior, which respond to external stimuli and release the incorporated compounds (cargos). Successful operation of logic requires a suitable design of sequences and the number of hybridized bases, as well as the operability of bifunctional aptamers without their mutual impact on the affinity of each other.

Another example of constructing logic OR and AND gates based on hydrogels and aptamers was the work in [107]. The hydrogel formed by photopolymerization was chemically modified with aptamers containing an NH_2_ group at the 3′- and 5′-ends. As a result, a hydrogel was obtained, the polymer chains of which were cross-linked with each other by means of aptamers. This made it possible to obtain a system that changes its physical properties in the presence of targets. To visualize the change in the system in the presence of targets, colloidal silicon nanoparticles were added to the hydrogel. For observation, the property of colloidal nanoparticles was used: the existence of photonic band gaps (PBGs) and the resulting structural colors. In the case of combining hydrogels and colloidal particles, one color was observed, while the addition of targets and compression of the hydrogel resulted in a change in the forbidden gaps (distance between nanoparticles) and, accordingly, the color. Ions of Hg ^2+^ and Ag^+^ were introduced as input data. The introduction of targets led to a change in the conformation of the aptamer and the formation of a complex between the aptamer and the metal. This was expressed in different degrees of compression of the hydrogel. In the absence of targets, the hydrogel with colloidal nanoparticles was red; the presence of one of the targets resulted in an average degree of compression of the gel, the color of which became yellow; the presence of both targets resulted in a high compression ratio of the gel, and its color turned green. The output signal of the system was a colorimetric signal. Depending on the magnitude of the shift in the reflection length of the hydrogel, threshold values were set for the logic gates: OR (shift more than 30 nm in the presence of one of the targets or both) and AND (shift more than 60 nm, occurs in the presence of only both targets). The concentration of targets for the execution of the logic would be defined as 0.1 μM, and the concentration of aptamers for gel modification: 0.2 mM for the Hg^2+^ aptamer and 0.5 mM for the Ag^+^ aptamer.

### 2.4. Logical Gates Based on Graphene Oxide

Oxide graphene, as with graphene, is a nanomaterial with unique electronic, thermal and mechanical properties [119]. It can serve as a carrier for the adsorption of biomolecules, since it has a large surface area with a π-conjugated structure [120]. It was found that ssDNAs are strongly adsorbed on a graphene oxide, while dsDNA cannot bind stably with it [121,122]. This property has been used to desorb ssDNA from the surface of graphene oxide by introducing complementary DNA or a target in the case of an aptamer [123]. Sorption of fluorophore-containing DNA on graphene oxide leads to fluorescence quenching due to the LrRET (long-range resonance energy transfer) [80,81], while in the case of DNA desorption, the fluorescence of the label is restored. These properties of graphene oxide are actively used to detect the desorption of aptamers in the presence of targets causing it.

The main strategy from the point of aptamer action is switching the aptamer from the surface of graphene oxide to a complex with a target [64,76,77]. Colorimetric and fluorescent signals are the main output signals in the logic gates. Different fluorescent labels (FAM, ROX, quantum dots) were restored by complexation of a target with aptamer from the graphene oxide surface [76,77]. Using various fluorescent labels (quantum dots), a half-adder and a half-subtractor were implemented, where each logical function had its own fluorescent channel [64]. Also, incorporation of hemin into the graphene oxide made it possible to obtain catalytical active material which was able to oxidize 3,3′,5,5′-tetramethylbenzidine [70]. This was used as a colorimetric output signal of the logic gate. One of the difficulties of working with graphene oxide is its separation from the aptamer solution after modification or working solution due to the low density of graphene nanosheets.

This section discusses the restoration of fluorescence after desorption of labeled aptamers from graphene oxide sheets upon interaction with targets [76]; a similar scheme using different fluorescent labels is shown [77]; there is a logic on hemin-containing graphene nanosheets capable of catalyzing a peroxidase-like reaction, while the interaction of aptamers with targets led to the aggregation of nanosheets among themselves [70]; a half-adder and half-subtractor based on aptamers labeled with quantum dots and sorbed on graphene oxide were developed [64]. Another logic gate was created using dsDNA staining with a fluorescent dye [124].

**Figure 9:**
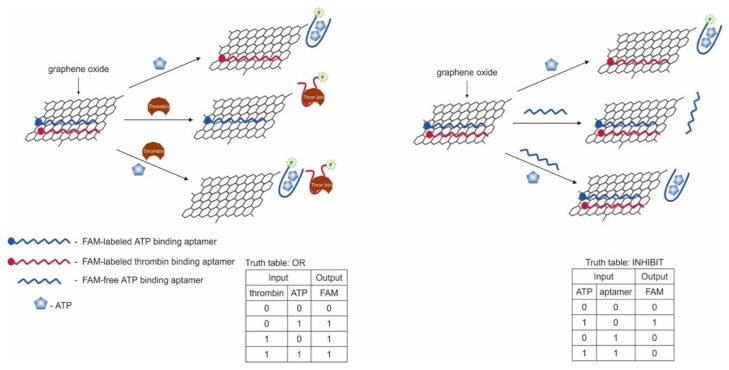
Logic gates OR and INHIBIT. Adapted from Reference [76]. ATP = adenosine triphosphate. Copyright © 2020 American Chemical Society.

It is known that the construction of logic gates [76] can be done using ATP aptamer [47] and thrombin aptamer [45]. The targets—ATP and thrombin—served as input signals, and fluorescence was the output signal (Figure 9). The aptamers contained a fluorescent FAM label and were adsorbed on a graphene oxide, which quenched the fluorescent label. In the presence of the target the labeled aptamer binds with it, which led to conformational changes that disrupt the interaction between DNA and graphene oxide. As a result of conformational changes, a fluorescent signal from the FAM-labeled aptamer appeared. Thus, the logic gate OR was constructed; the presence of thrombin and ATP together and separately caused the appearance of a signal. Output signal 1 was in the presence of ATP > 2 μM and/or thrombin > 0.04 nM. When ATP and its unlabeled aptamer (two input signals) were present in the system, the INHIBIT gate was implemented. The INHIBIT gate was implemented in a similar manner in the presence of thrombin and its unlabeled aptamer. The combination of approaches in OR and INHIBIT gates leads to discrimination of the signal from a specific target, when it is not known which target/targets were present in the solution. The threshold concentrations for signal 1 when the valves are aligned are > 3 μM of ATP and > 0.1 nM of thrombin. The approach is interesting in terms of the potential of sensory capabilities. The OR gate can be used to detect the presence of different targets in the same sample, although it will not be known which target triggered the signal. This can be overcome by using various fluorescent labels and creating high-performance fluorescent imaging systems. Also, the use of long aptamers (>29 np, aptamer for thrombin) can become a problem, since in this case the fluorescent label can remain quenched even when bound to the target. The logic realization time was 30 min.

It is also known the construction of AND logic gate based on aptamers for thrombin and ATP and graphene oxide [77]. FAM-labeled aptamer for thrombin and ROX-labeled aptamer for ATP were adsorbed on the surface of graphene oxide. In the absence of targets, the fluorescence of the labels was quenched due to the proximity of the graphene oxide surface. The introduction of targets into the system led to the formation of a target–aptamer complex and the restoration of the fluorescence due to the desorption of the aptamer from the surface of the carrier. The detection limit for thrombin and ATP was 1 nM and 10 μM, respectively. A high change in fluorescence (F-F0) of both labels FAM and ROX was defined as output 1, while no fluorescent change in FAM and/or ROX was 0. Thus, an AND gate was constructed using targets: thrombin and ATP. Fluorescence measurements time is 150 min.

**Figure 10:**
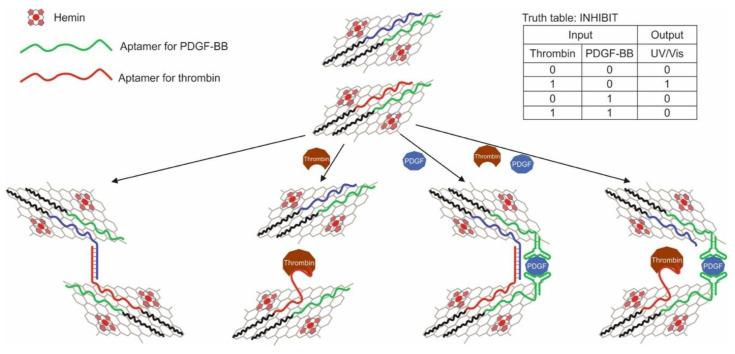
Logic gate INHIBIT. PDGF = platelet-derived growth factor. Adapted from Reference [70]. Copyright © 2020 American Chemical Society.

Another work considers the logic OR and INHIBIT built on hemin-containing graphene nanosheets (nanosheets) [70]. Hemin-containing graphene has been obtained by wet chemistry [125]. This material possesses peroxidase properties and is capable of oxidizing TMB (3,3′,5,5′-tetramethylbenzidine) in the presence of H_2_O_2_ with the color change of the solution. Modified graphene, as with ordinary graphene, is capable of adsorbing DNA due to π–π stacking and hydrophobic interaction [120,121,122]. Thus, to implement the OR logic gate, ssDNAs containing aptamers for thrombin and PDGF-BB were applied to the surface of hemin-containing graphene nanosheets; in addition to aptamers, ssDNAs contained a 20 bp tail for binding with graphene in the presence of targets. In this case, in the final experiment, a mixture of nanosheets with various applied ssDNAs was studied. We used two aptamers for thrombin, binding different parts of thrombin, and one aptamer for PDGF-BB, which is also capable of interacting with two different regions of the target protein. In the implementation of the OR gate, the addition of thrombin and PDGF-BB separately and together resulted in the aggregation of graphene nanosheets. As a result, after centrifugation, fewer unaggregated nanosheets remained in solution, capable of catalyzing the peroxidase reaction, which was responsible for the output signal of the logic. The decrease in the colorimetric signal (λ = 652 nm) from the reaction was taken as 1 in this case. Aggregation occurred due to the interaction of some aptamers from one nanosheet with protein targets and other aptamers from another nanosheet with the same protein targets, but different binding sites. A limitation of this sandwich scheme is that targets with two binding sites must be used. To overcome this limitation, the authors proposed a competitive scheme using a protein target and DNA complementary to aptamer-containing DNA as inputs. In addition, the INHIBIT logic gate was designed (Figure 10). It used three DNA adsorbed on the graphene surface: one nanosheet—aptamer for thrombin, an aptamer for PDGF-BB, another nanosheet, complementary to the aptamer for DNA thrombin, the same aptamer for PDGF-BB. Only the addition of thrombin resulted in an output signal of 1, which signified less aggregation of nanosheets and corresponded to the high colorimetric signal of the peroxidase-like reaction after centrifugation. The absence of targets led to aggregation due to hybridization of the aptamer to thrombin with complementary DNA; in the presence of PDGF-BB, aggregation occurred due to the double interaction of the protein target and aptamers to it. The logic action time was 12 h; observation of the peroxidase reaction was ~20 min.

**Figure 11:**
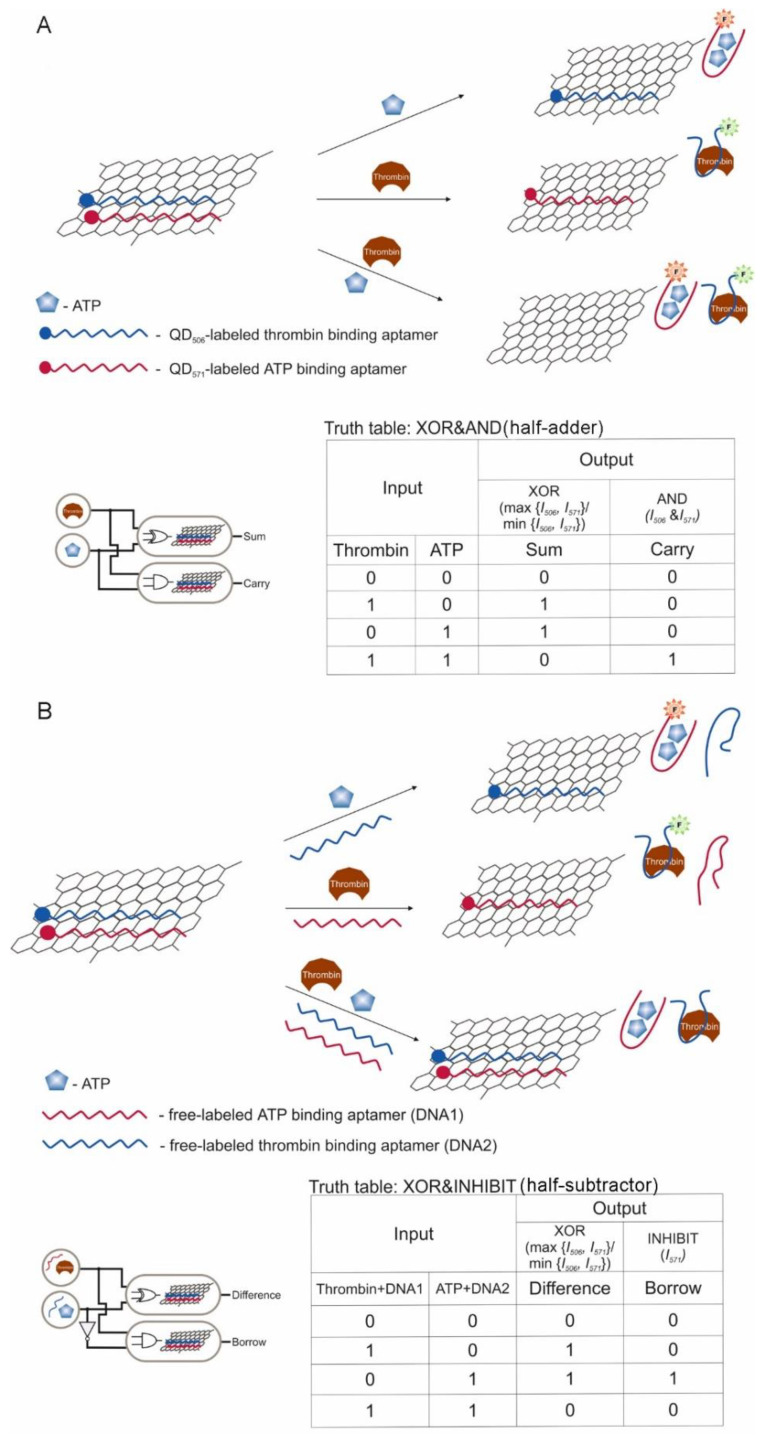
Half-adder (**A**) and half-subtractor (**B**) functions. ATP = adenosine triphosphate. Adapted from Reference [64] by permission of The Royal Society of Chemistry.

Using a combination of quantum dot (QD), aptamers and graphene oxide, logic gates have been developed to implement half-adder (Figure 11A) and half-subtractor (Figure 11B) functions [64]. Various QDs were used as fluorescent labels for aptamers: QD_506_ (λ = 506 nm) for the thrombin aptamer and QD_571_ (λ = 571 nm) for the ATP aptamer. Originally, the half-adder, which combines the XOR and AND gates, was built using ATP and thrombin as two inputs. Adsorption/desorption of labeled QD aptamers from graphene oxide led to a fluorescent response of the system. The XOR gate was achieved by determining the ratio of fluorescence intensity between QD_506_ and QD_571_ (max {I_506_, I_571_}/min {I_506_, I_571_}) as the output signal Sum, with the value 2 as the threshold value above which the output 1, a below 0. AND gate can be achieved by determining the fluorescence intensity of QD_506_ and QD_571_ at two wavelengths as the Carry. Output 1 was determined when the intensities I_506_ and I_571_ were simultaneously higher than the threshold value 2, in the opposite case-0. By changing the inputs, the proposed system made it possible to compose a logical function half-subtractor. Thus, the inputs to this logic were combinations of thrombin with an unlabeled aptamer for ATP and ATP with an unlabeled aptamer for thrombin. The half-adder combines an XOR gate and an INHIBIT gate in parallel to generate Difference and Borrow output, respectively. The XOR outputs were defined in the same way as before for the half-adder. The INHIBIT gate was implemented by detecting the fluorescence intensity of one of two QDs, for example, I_571_, as an output with the same threshold as before. In addition to the compiled logical operations, the researchers showed the possibility of reusing graphene oxide with aptamers containing QD. This turned out to be possible by changing the pH of the solution from 8.0 to 6.0, since with a decrease in pH, the charge of guanines in the composition of aptamers is neutralized, and they are not able to form G-quadruplexes for binding with thrombin and ATP. This led to the release of targets from the complexes and the formation of ssDNA. The logic implementation time was 40 min.

As with the previous gates, an AND gate was created, where ATP and ssDNA were used as inputs [124]. On the surface of graphene oxide, a DNA duplex containing an aptamer on ATP and a complementary sequence was physically immobilized through the single-stranded end. The output signal of the system was the fluorescence obtained from the addition of the PicoGreen dye, which stains dsDNA. Signal “1” was formed only under the condition of the combined addition of ATP and ssDNA, since in this case a duplex was formed between the introduced ssDNA and the sequence dehybridized from dsDNA due to the formation of a complex of the aptamer with the target. The range of ATP detection was 20–400 nM, the detection limit was 142.6 pM.

## 3. Electrochemical Detection

Electrochemical detection is one of the main detection methods in biosensors based on aptamers [126,127,128]. In addition, using electrochemical detection, several aptamer-based logic gates were obtained.

According to the organization, the existing logic gates can be divided into biofuel cells [129,130,131] and two-/three-electrode cells [43,53,78,132,133,134].

The difference between them is that the former contains redox enzymes that use a substrate (fuel) to generate an electrical signal. In this case, the system consists of a cathode and an anode, which, as a rule, are spatially separated and modified by enzymes and aptamers. In such systems, aptamers are used for protein targets, which, when complexed with aptamers, create restrictions on the diffusion of substrates to enzymes and signal transmission from the enzymatic reaction to electrodes [129,130]. Also, an aptamer for a low-molecular-weight target (AMP) as part of ssDNA containing a peroxidase-like DNAzyme was used [131].

The second, and more common, are two-/three-electrode cells with a modified working electrode (gold, graphene oxide) on which the aptamer–target complex is formed. The formation of a complex leads to a change in the electrical signal (current, potential, etc.) due to the appearance of an electroactive compound or a catalytic label near the electrode surface. As electroactive labels the use of methylene blue [132], ferrocene [53] and [Ru(NH_3_)_6_]^3+^ [78] have been shown. The catalytic labels were peroxidase-like DNAzyme [43] and peroxidase [133], which catalyze the reaction with the participation of an organic substrate and hydrogen peroxide. The approach/distance of the mark to/from the electrode surface led to a change in the signal.

Also, the features of logic gates with electrochemical detection, in contrast to other types of detection, is that ssDNAs (including aptamers) are often used as input data along with targets for aptamers [43,132,134] or enzymes [53,131]. A logic gates INHIBIT [43,53,131] and NAND [78,129,133] are more common in comparison with other gates within this type of detection.

Separately, it is worth noting a logic operation based on a nanofluidic channel with an aptamer on ATP, which is capable of performing the ON–OFF logical operation [134]. The output signal is the ionic current in the channel, which can be “switched on” or “switched off” when a target or two ssDNA is introduced, one of which contains an aptamer.

### 3.1. Two-/Three-Electrode Cells

#### 3.1.1. Electroactive Compounds

This section describes logic gates containing an aptamer [132] immobilized on the surface of a gold electrode and a part of the aptamer [53] with electroactive labels, which, when forming a complex with targets, approach the electrode surface. One of the gates uses an enzyme as an input to degrade the target for the aptamer [53]. It is also known as a system consisting of a graphene electrode with physically immobilized aptamers and an electroactive compound [Ru(NH_3_)_6_]^3+^; the introduction of targets led to the desorption of aptamers from the surface [78].

**Figure 12:**
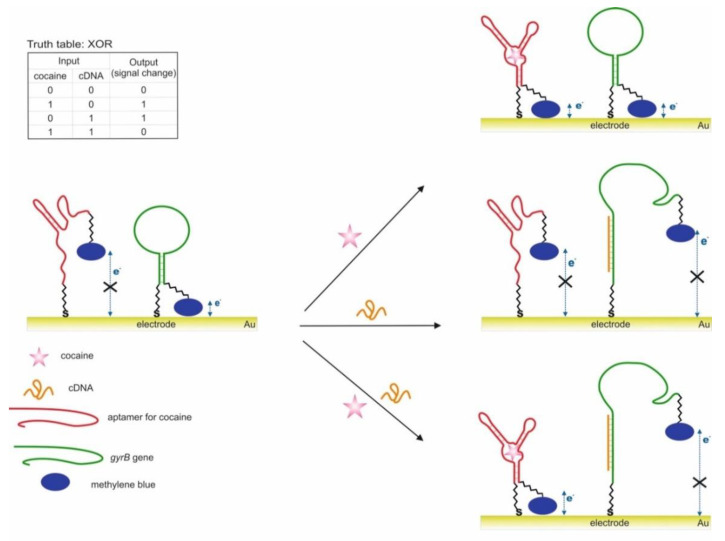
Logic gate XOR. Adapted from Reference [132]. Copyright © 2020, American Chemical Society.

A logic system on gold microelectrodes with an XOR gate was composed, where the reduction current was taken as the output signal [132] (Figure 12). On the surface of the electrode were immobilized two ssDNAs containing at the other end of the redox label methylene blue [50,98,135,136]. One of the ssDNA is the aptamer for cocaine [49,50], and when the aptamer interacts with the target, methylene blue becomes closer to the electrode, which leads to the direct of electron transfer. Another ssDNA is an oligonucleotide sequence in the form of a hairpin, the end of which with a redox compound is located near the surface of the electrodes, and electrons are exchanged between methylene blue and the electrode. Based on this system, the XOR logic was composed. As targets/input data cocaine and ssDNA were used; ssDNA was complementary to the hairpin DNA and straightened it so that the redox compound moved away from the surface. The output signal was taken as 1 in the presence of cocaine > 250 μM and ssDNA > 50 nM; lower concentrations corresponded to output 0. In this case, a signal change of more than 5% was assigned as 1, and less than 5% as 0. According to the same principle, a three-input valve was composed with the addition of urea as a third component that unfolds structured DNA. A urea concentration of >1 M was determined as the output 1, a concentration below as 0. The described logical approach is interesting; however, it has low sensitivity due to the insignificant change in the signal of ~2–25% between states 1 and 0. This is also confirmed by the high concentrations of targets used to generate the output signal 1.

The example of constructing a logic gate based on a split aptamer can be found in [53]. The authors used a cleaved aptamer on ATP [47] so that one of its fragments was immobilized through a disulfide bond on the surface of the gold electrode, while the other free fragment was in solution and contained ferrocene at one of its ends. When ATP was added to the system, a stable complex with it and the split parts of the aptamer was formed, while the ferrocene label was located closer to the electrode surface. It should be noted that the electrode surface (not occupied by the aptamer fragment) was passivated with 1-undecanethiol to minimize the direct transfer of electrons from the electroactive compound to the electrode surface. Initially, ATP and the enzyme adenosine deaminase were used as input data. Adenosine deaminase catalyzes the conversion of ATP to inosine triphosphate (ITP) with which the aptamer does not bind. The current from the oxidation of an electroactive compound, ferrocene, was taken as an output signal. On the basis of these compounds, the INHIBIT gate was built: the output signal taken as 1 was only in the case of the presence of one ATP target; the joint addition of the enzyme and ATP can lead to the degradation of the aptamer complex with the target [137]. To implement the logic gate, high concentrations of ATP and enzyme were used: 10 μM and 0.03 U/mL, respectively. The current value for these concentrations was taken as the threshold value for determining the output 0 and 1. It should be noted that the value of the signal obtained in this system depends on the density of the aptamer and the ferrocene fragment: low density strongly affects the sensitivity and applicability of the system (large fluctuations in the output signal). Therefore, the authors suggested introducing into the medium one more electroactive compound, ferrocyanide (K_4_[Fe(CN)_6_]), which is in solution in a free state. According to the value of oxidation–reduction potentials, ferrocyanide transferred electrons to ferrocene, which in turn to the electrode. This approach led to the creation of the INHIBIT–AND gate based on three inputs: ATP, enzyme and K_4_[Fe(CN)_6_]. As a result, it was possible to reduce the detection limit of ATP compared to the previous scheme 130 times greater: from 1 μM to 7.5 nM. The detection limit for enzyme activity was 0.002 U/mL. The signal taken as 1 corresponded to the current value of 1.5 μA (when ATP is ≥10 μM and the enzyme activity is <0.01 U/mL). The signal taken as 0 was less than 0.1 μM (when ATP was <0.1 μM and the enzyme activity was ≥0.03 U/mL). The addition of the third component increased the difference between 0 and 1 to 15 times, which made it possible to better discriminate between signals from certain concentrations of the enzyme and ATP. Freely in solution, an electroactive compound allows the constant transfer of electrons to the mediator (ferrocene) and thus amplifies the signal, linearizing it depending on the concentration of other input data. The XOR gate was compiled with the addition of the fourth component, K_2_IrCl_6_, which is responsible for the onset of the cathodic current, and also allows more accurate confirmation of the signal from ATP and the enzyme and the determination of 0 and 1. However, when K_4_[Fe(CN)_6_] and K_2_IrCl_6_ are added together, the output signal taken as 0, since the current decreases significantly in this case due to the occurrence of cross processes in the solution. The XOR gate can be used to confirm the results (signal 1 if there is ATP in the system and one of the free electroactive compounds) and eliminate inaccurate diagnoses caused by a system malfunction. The approach implemented by the authors is quite interesting; at the same time, the possibility of its extension to other targets depends in the performance of their split aptamers. The logic implementation time was above 30 min.

The NAND logic gate based on graphene oxide electrodes was described [78]. For this, a bifunctional aptamer was physically sorbed on the graphene-modified electrode. Then the electrode was placed in a solution of the electroactive compound [Ru(NH_3_)_6_]^3+^, which was also sorbed due to electrostatic interaction with the negatively charged phosphate backbone of the aptamer. Thrombin (5 mg/mL) and lysozyme (15 mg/mL) were introduced into the system as input data. The manufactured logic aptasensor could detect the presence of both targets by means of the built-in NAND logic gate. The thrombin aptamer and the lysozyme aptamer were combined into one ssDNA, which is a bifunctional aptamer. Such bifunctional aptamer on the graphene surface could capture the target from the solution, which led to a decrease in the electrochemical signal from the surface-bound electroactive compound [Ru(NH_3_)_6_]^3+^. Logic output is the peak current during differential pulse voltammetry. Changes in current signals greater than 50% and less than 50% were defined as 0 and 1, respectively. Thus, only the simultaneous presence of both targets led to a change in the current by more than 50%, since in this case the bifunctional aptamer in the complex with targets was desorbed from the graphene surface (the peak current decreases by 75% from the initial state of the system). This led to the desorption of [Ru(NH_3_)_6_]^3+^. The logic action time, incubation in a solution with targets, was 60 min. The linear range and the calculated detection limit of thrombin by the proposed aptasensor was 0.5 ng/mL–5 mg/mL and 0.15 ng/mL, respectively. Likewise, the linear range and calculated detection limit of lysozyme by the sensor was 3 ng/mL–15 mg/mL and 1.2 ng/mL.

#### 3.1.2. Catalytic Label

This section shows the use of a bifunctional aptamer hybridized with a sequence containing DNAzyme [43]. There is a gate with an immobilized bifunctional aptamer on the surface of a gold electrode, its complementary DNA contains biotin, through which streptavidin-containing peroxidase binds [133].

**Figure 13:**
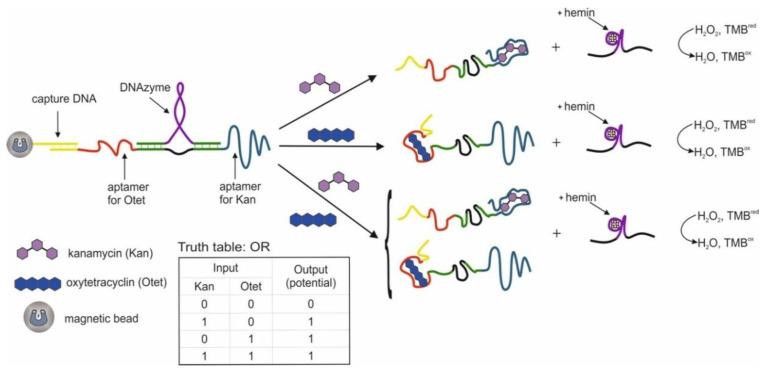
The OR logic gate. TMB = 3,3′,5,5′-tetramethylbenzidine hydrochloride. Adapted from Reference [43]. Copyright © 2020, American Chemical Society.

The implementation of the OR and INHIBIT algorithms is shown using a bifunctional oligonucleotide containing aptamers on kanamycin [138] and oxytetracycline [139] in its side parts [43]. The bifunctional oligonucleotide partially hybridized with a DNA probe bound to magnetic particles through the biotin–streptavidin complex, as well as with DNzyme, which catalyzes the peroxidase oxidation of 3,3′,5,5′-tetramethylbenzidine hydrochloride in the presence of hemin (Figure 13). The DNAzyme was dehybridized from the bifunctional oligonucleotide in the presence of each target separately and together and served as a label for the chronopotentiometric detection. The optimal incubation time of modified magnetic particles with targets was 60 min, after which the particles were separated, and hemin for DNAzyme was added to the supernatant. Furthermore, the determination was carried out using an ion-selective electrode, the potential of which changed during the DNAzyme reaction. The logical function OR was performed when kanamycin (threshold concentration 10 nM) and oxytetracycline (threshold concentration 10 nM) were added separately or together, the signal was taken as 1 in this case. The detection limit and linear range for kanamycin are set equal to 7.5 nM and 10–100 nM, and oxytetracycline, equal to 9.8 nM and 10–100 nM, respectively. The logical function INHIBIT was implemented using two free aptamers and their targets as input. As a result, the output signal 1 was only when the antibiotic was introduced into the system; when the antibiotic and the free aptamer were administered together, the output signal was 0, since the free aptamer bound to the antibiotic, which did not interact with the bifunctional oligonucleotide in this case. The authors also proposed a combinatorial approach to identify a specific antibiotic that is present in a sample. The approach overlaps with the previously described approaches [65]. Its possible limitation for widespread use with various targets may be the impossibility of constructing a bifunctional aptamer based on long-chain single aptamers. Also, it is not entirely clear why DNAzyme dissociates from the sequence with aptamers. Since the link between the sequence and the DNAzyme and the sequence with the aptamers is strong enough (9 and 11 nucleotides on both sides of the DNAzyme), the aptamers themselves remain unhybridized. The authors presented calculations of the energies of interaction of aptamers with targets and the energies of complementary sequences, according to which they suggest dehybridization. Dehybridization was also confirmed by PAAG electrophoresis data.

**Figure 14:**
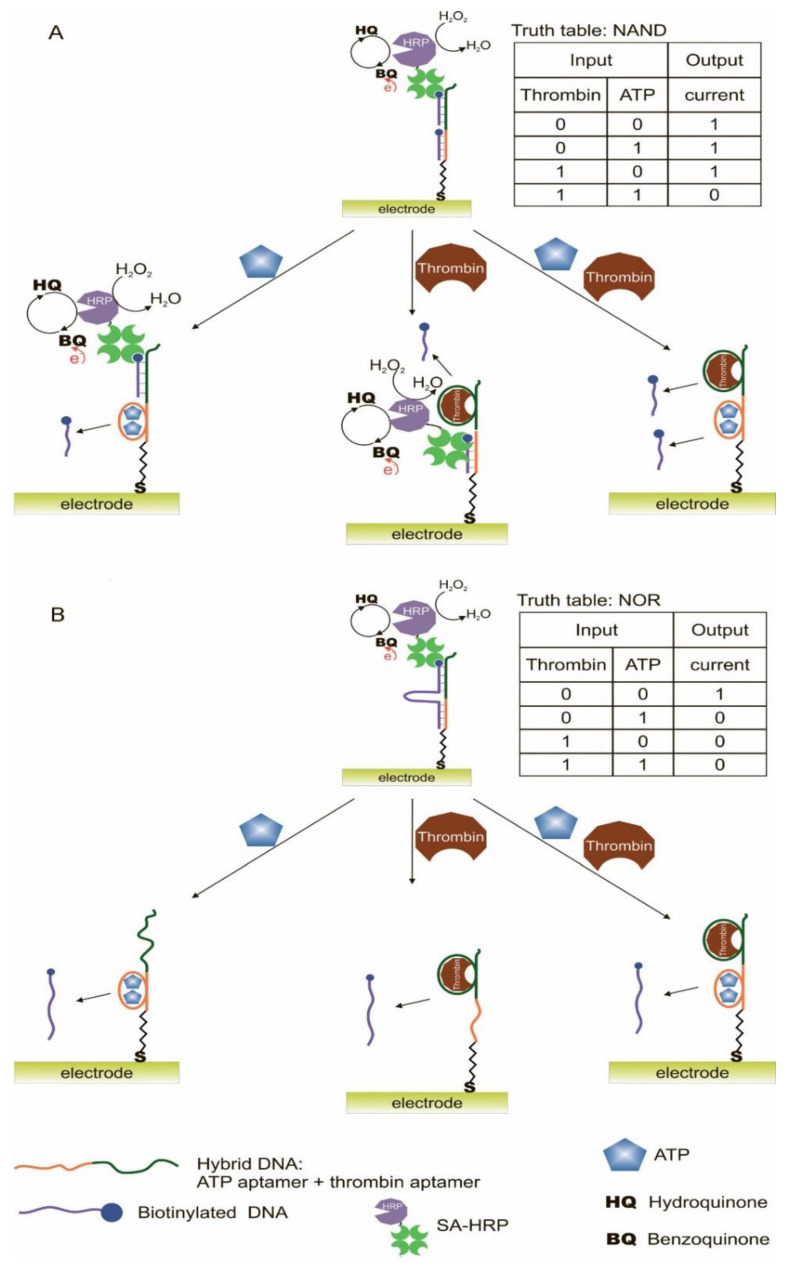
(**A**)—Logic gate NAND. (**B**)—Logic gate NOR. ATP = adenosine triphosphate, SA-HRP = streptavidin-horseradish peroxidase conjugate. Adapted from Reference [133]. Copyright © 2020 Elsevier B.V.

Also, based on split [56,98] and linked aptamers against thrombin and ATP, a series of OR, AND, NOR and NAND (Figure 14) logic gates was constructed [133]. The mechanism for constructing partially coincides with that described in the previous study. In this case, the aptamers were immobilized on the electrode surface, and the output signal was the current associated with the peroxidase serving as a label. The OR gate was organized using two hybrid DNAs, each containing a part of the aptamer to ATP and thrombin. One of the DNA was immobilized on the surface of the electrode, the other was in a free state and contained a biotin label. In the presence of one or both targets, both hybrid DNAs were collected near the electrode surface due to the aptamer–target interaction. The addition of streptavidin-containing peroxidase led to its binding to biotin. Immobilized peroxidase oxidized hydroquinone to benzoquinone in the presence of H_2_O_2_, and benzoquinone, in turn, was reduced electrochemically on a gold electrode. The output signal, taken as 1, was 0.4 μA and corresponded to 80 μM of ATP and 64 nM of thrombin. The optimal assembly time for aptamers was 40 min, the blocking of non-specific adsorption was 15 min and the incubation time with modified peroxidase was 30 min. The logic AND gate implied the use of three aptamer fragments: a hybrid DNA, composed of two parts of aptamers for ATP and thrombin, and the two remaining parts of aptamers, one of which contained a biotin tag. The output signal appeared only when two targets were present simultaneously in the system. The logical NOR gate was implemented using a hybrid DNA containing both aptamers and a partially complementary sequence containing biotin. Thus, in the absence of targets, the electrode reduction current was higher than 0.4 μA, and the output signal was taken as 1. The introduction of one or both targets led to the dissociation of the complementary sequence from the hybrid DNA and the formation of target–aptamer complexes. 

### 3.2. Biofuel Cells

Biofuel cells (BFCs, [140])—cells with self-regulating energy release as defined by the creators—are a special kind of system that uses enzymes or microorganisms that convert biofuels into an electrical signal [141]. The biofuel cell is based on an electrode system consisting of a cathode and an anode. The surface of the electrodes is modified with biocomponents: proteins, enzymes, and aptamers. In general, biofuel cells contain many other different components inside the cell: substrates, electroactive compounds, and modifiers. Processes that occur with biocomponents and compounds in solution lead to the closure/opening of the electrical circuit and the occurrence of a certain level of current between the cathode and the anode.

Logic gates based on a biofuel cells and aptamers are systems in which the interaction of the aptamer and the target serves as a kind of switcher and leads to a change in the electrical signal of the cell. 

This section describes a biofuel cell with an enzyme-modified anode and cathode and a common reaction solution [129]; there is a system with a divided space between the cathode modified by the bifunctional aptamer and the unmodified anode with the enzyme in the anode space [130]. Another example is a separated cathode and anode space, where the cathode is modified with ssDNA with an aptamer and DNAzyme, and the anode is modified with an enzyme [131].

**Figure 15:**
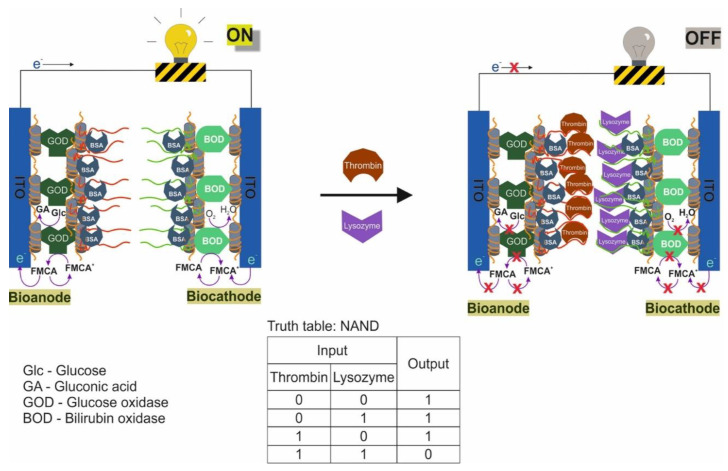
Logic gate INHIBIT. Adapted from Reference [129]. Copyright © 2020, American Chemical Society.

The first BFC with an integrated logic NAND algorithm was manufactured by the authors in [129]. The proposed algorithm was implemented using aptamers for thrombin [44] and lysozyme [46] and a logical system built on them, which determined whether both targets were present simultaneously in the sample (Figure 15). The BFC consisted of an anode and a cathode made of indium tin oxide. A layer of poly(diallyldimethylammonium chloride) with carbon nanotubes (PDDA-CNTs), the enzyme glucose oxidase (for the anode, the enzyme oxidizes glucose with the release of oxygen) and bilirubin oxidase (for the cathode, the enzyme reduces oxygen to water), aptamers for thrombin (anode) and lysozyme (cathode) and bovine serum albumin. The reaction medium contained glucose as a substrate for the enzyme and ferrocene monocarboxylic acid as an electroactive compound. Output 1 was set when the open circuit voltage was greater than 0.05V; output signal 0 was set when the open circuit voltage was below 0.05 V. An output signal of 0 appeared only when both targets were present in the system, thrombin (cut off concentration 8 nM) and lysozyme (cut off concentration 20 nM), interacting with aptamers; as a result, spatial restrictions were created near the surface of the electrodes for the free action of enzymes and diffusion of the electron mediator—ferrocene monocarboxylic acid. The potential difference between the electrodes decreased significantly. This approach is most likely applicable only to large protein targets that can bind to their aptamers and effectively block the transfer of electrons to the surface. It should be said that this logical system is disposable.

The above approach with electrodes made it possible to implement only a single NAND gate. Further research led to the creation of logic based on gold microelectrodes with the possibility of reusability and integration into the microfluidic system [130]. The surface of the microelectrodes was modified in such a way that an ON/OFF switcher was implemented. In the system, the anode and cathode spaces were separated by a Nafion membrane. The cathode was modified with an SH-containing oligonucleotide sequence, with which the bifunctional aptamer partially hybridized. A bifunctional aptamer was ssDNA sequence containing aptamers for thrombin and ATP. In addition, in the cathode space there was an oxidizing agent, K_3_[Fe(CN)_6_]. The anode was not modified, and the anode space contained glucose oxidase, which oxidizes glucose in an oxygen-saturated solution, and an electroactive compound—ferrocene monocarboxylic acid. The output signal was the current density at the cathode, the threshold value was 75 μM/cm^2^. In the absence of thrombin and ATP, the system was turned ON. The addition of ATP to the cathode region resulted in the binding of the aptamer to ATP and dehybridization of the bifunctional aptamer from the electrode surface, which also corresponded to the ON state of the system. The addition of thrombin led to blocking of the electrode surface for the electroactive compound due to its binding to its aptamer; dehybridization did not occur in this case. The state of the system was assessed as OFF. The combined addition of ATP and thrombin, which also leads to dehybridization of the bifunctional aptamer from the surface, corresponded to the ON mode. This was the essence of the RESET function, which can be implemented by adding ATP to the system. The authors also showed multiple (up to 10 times) system switching. The logic implementation time (from the introduction of targets to the production of the output signal) was 120 min.

Another approach within biofuel cells is presented when implementing the INHIBIT gate [131]. In this case, the cathode was modified with ssDNA which contained the DNAzyme sequence with peroxidase activity and the aptamer sequence for adenosine monophosphate (AMP). The ssDNA was in the hairpin configuration, and the DNAzyme and aptamer were blocked in the duplex part of the hairpin. The anode was modified with a composite of glucose dehydrogenase, carbon nanotubes and Meldola blue (used as a mediator). Glucose, a substrate oxidized in the presence of NAD^+^, was used as a “fuel”. The cathode and anode were separated by a Nafion membrane. When AMP was added to the cathode space, the aptamer formed a complex with it. As a result, the hairpin opened, and in the presence of hemin, an active DNAzyme was formed, catalyzing the reduction of H_2_O_2_. The output signal of the system was the current. To demonstrate the logic, adenosine aminase was used, which catalyzed the conversion of AMP to inosine monophosphate. AMP and enzyme served as input data. Thus, output signal 1 in the INHIBIT gate was realized only in the presence of AMP: the enzyme alone did not activate DNAzyme, and the joint presence of the enzyme and AMP resulted in the formation of a product that did not bind to the aptamer. It should be added that the presented logic gate based on DNAzyme can work in the absence of an external power source. In this case, the output power is reduced by 5% in 20 min of operation and by 30% in 40 min of operation. The logic implementation time (from the introduction of targets to the receipt of the output signal) was 120 min.

### 3.3. Other

Researchers have developed a biohybridic nanofluidic channel with an ATP aptamer that can perform the logical operation IMPLICATION (if… then), ON–OFF mode [134]. Initially, alumina nanochannels were chemically modified with 5′-aminated DNA (35 bp), which included an aptamer on ATP. Then two others were added to these DNAs, partially complementary to each other: P1 and P2. P2 also included an aptamer on ATP. As a result, a super-sandwich structure of DNA was formed inside the nanochannel containing several P1 and P2 units attached to the aminated DNA on the channel walls. When ATP is added to the system, the aptamers bind to the target and the super-sandwich structure is destroyed. After assembling the super-sandwich, the ion current in the channel drops sharply from 6.2 × 10^−5^ A (ON state) to 1.1 × 10^−10^ A (OFF state) at +200 mV. The ionic current is restored to 4.9 × 10^−5^ A after treatment with 1 mM ATP. Super-sandwich assembly and ATP-coupled super-sandwich degradation in the nanochannel (average channel width 60 nm) showed asymmetric response times of about 500 min (ON–OFF) and 100 min (OFF–ON). The logic operation IMPLICATION uses ssDNA (P1 and P2, 1 μM) and ATP (1 mM) as inputs and a change in transmembrane ion current as an output signal. For the output, the change in the signal by more than 100 times (with respect to the state when the nanochannels are modified only by aminated DNA) was defined as 1, otherwise, as 0. It can be seen that a distinct output signal 1 appears if and only when both ssDNAs are present in the system and ATP is absent. The described work is, according to the authors, the first work on the creation of a logical operation based on nanofluidic channels.

## 4. Cell-Based Logic Gates

The surface of cell membranes has a unique set of proteins, lipids and carbohydrates that are involved in cell growth, proliferation and signaling [142,143]. Changes in the level of expression and the set of cellular receptors can lead to systemic dysfunction, as happens with cancer cells [144,145].

Development of the cell-SELEX method [146] led to the production of a whole set of aptamers to cell membrane receptors [147,148]. These aptamers have demonstrated the ability to identify different patterns of expression of membrane receptors in various cell types [149]. They can also differentiate the same cancer cell population at different stages of the cell cycle [148].

The main purposes of using logic gates with the participation of aptamers and cells are:

- differentiation (imaging) of cells of a certain type among the general set of cells; most often it is necessary for an accurate diagnosis;
- implementation of targeted delivery of the effector to a specific type of cells; for example, an activator in the case of lymphocytes [36], or a drug that stops the proliferation of cancer cells [36,40].

The input data of such logic gates are cells, or rather, surface markers. Programmable analysis of multiple markers will allow the development of a complex disease profile which will lead to a more accurate diagnosis and further possible intervention. For example, in diseases such as leukemia, both in healthy and in sick subpopulations of white blood cells, surface markers are indistinguishable in the analysis of one receptor; therefore, the simultaneous determination of several surface receptors allows for the increase in the diagnostic accuracy in the differentiation of similar cells [150,151] and imaging of cell samples for analysis to biomedical personnel.

Structurally, the logic built on cells and aptamers, as a rule, implies the participation of a logic “robot” consisting of an oligonucleotide backbone as a framework [32,34] of several structurally switchable aptamers as “capture toe” and a logically controlled DNA duplex as “effector toe”. The gripping fingers perform two functions: first, the aptamer interacts with a cellular marker on the surface, and then the corresponding bar code oligonucleotide is generated to activate the effector. Finally, the “effector” analyzes these oligonucleotides and autonomously decides when generating a diagnostic signal (such as fluorescence) and a therapeutic effect. The gripping fingers function is achieved by structurally switchable aptamers.

There are useful publications partly describing some of the logical operations involving cells and aptamers [152,153]. In this section, attention will be focused directly on the logic gates’ organization which can provide scientific inspiration for further research in the field of aptamer logic, and also show the work that has been done to date.

If we evaluate the conducted work in retrospect, at first, logic gates were fabricated based on aptamers which were physical unrelated to each other [40]. However, in this case, false positive signals were received from neighboring cells. Then logical nanorobots [32,34], described above and containing the physically linked aptamers in a single design, were created. Next, near the cell’s surface, a hybridization chain reaction was carried out with the set of aptamers and fluorescent hairpins, which provided a brighter staining of labeled cells due to the number of joint labels [154]. Besides this, construction of logic gates using microelectrodes to determine the type of cells is known [33]. In these studies, aptamers against membrane targets of cancer cells are used.

Also, logic gates (YES, NOR, AND, OR and AND/OR) using aptamers on membrane receptors c-Met and CD71 are known [155]. Light and ssDNA are used as inputs, resulting in a logical assembly of DNA. As a result of the logic, the c-Met receptor and CD71 are in close proximity, which interferes with the ligand-receptor interaction of c-Met and suppresses its functions. This logic gate design is an example of a tool for modulating cellular signal transmission with the use of aptamers.

**Figure 16:**
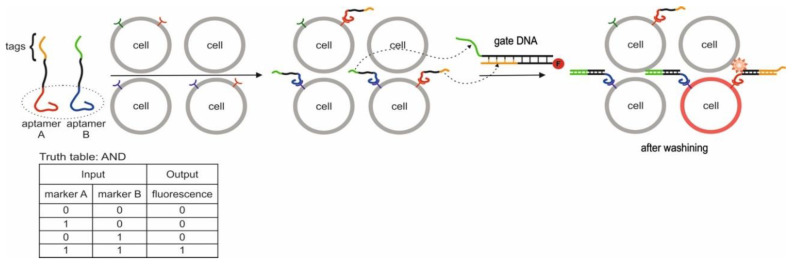
The AND logic gate. F = fluorescent label. Adapted from Reference [40]. Copyright © 2020 American Chemical Society. Further permissions related to the material excerpted should be directed to the ACS.

A logical analysis of 2–3 cell surface markers of cancer cells using aptamers has been proposed [40]. This approach made it possible to differentiate cells of one subpopulation when compared with cells of a similar type. The authors have compiled the logic gates AND, OR, and NOT. Several components were used in the implementation of logic gates. The first component was ssDNA containing aptamers that interacted with the membrane receptors of cancer blood cells. The range of aptamers studied by the authors is very wide: sgc8c, TD05, sgc4f, TE17, TE02. In addition to aptamers, each ssDNA contained a spacer and a fragment (tag) involved in subsequent cascade logic reactions using additional sequences. The second component of the system was ssDNA/dsDNA (gate DNA), which contains either a fluorescent label/photosensitive compound and triggers toehold-mediated strand displacement reactions with the participation of tail fragments of ssDNA with aptamers. These tail fragments served as a kind of bar code for further operations. It should be noted that ssDNA with aptamers are not physically connected with each other and the main logic gates (AND, OR, NOT) were carried out through their joint cascade action based on the design of tail fragments and dsDNA with labels: the released sequence initiated the next strand substitution and release new sequence. This led to the fact that if a cell had certain membrane receptors, it received a fluorescent label (Figure 16) or a photosensitive compound (chlorin e6, Ce6). Ce6 is used in photodynamic therapy [156,157]. The photosensitive compound generates reactive oxygen species under the influence of light, which act on the labeled cell, i.e., the output signals of the logic were fluorescence (fluorescence microscope and flow cytometry) and cell viability assessment using propidium iodide staining. The incubation time with aptamers was 30 min, then, after washing, incubation for 1 h with dsDNA. Integrated logic systems i1ANDNOTi2, i1ANDi2ANDi3, i1AND (i2ORi3), i1ANDNOT (i2ORi3), i1ANDi2ANDNOTi3, i1ANDi2ANDi3ANDi4, i1ANDi2ANDi3ANDNOTi4 were built based on AND, OR, and NOT gates. As a rule, in this case, the third component of the system was used, the assisting ssDNA, which in the free state participated in the cascade reaction of generating the output signal. However, in the presence of the corresponding membrane receptor in the cell and the associated aptamer, the assisting DNA hybridized with the tail fragment, and the output signal of the system was equal to 0. Thus, the NOT gate was realized. The proposed logic is the differentiation of high-order cellular markers. However, it is sometimes difficult to distinguish between positive (true) and negative (false) output signals due to the slow kinetics and incomplete circuit replacement. At the same time, during differentiation of cells in the general population, false positive signals from membrane receptors of neighboring cells may occur, since, as noted earlier, ssDNA with aptamers are not physically linked to each other, but are operatively linked.

**Figure 17:**
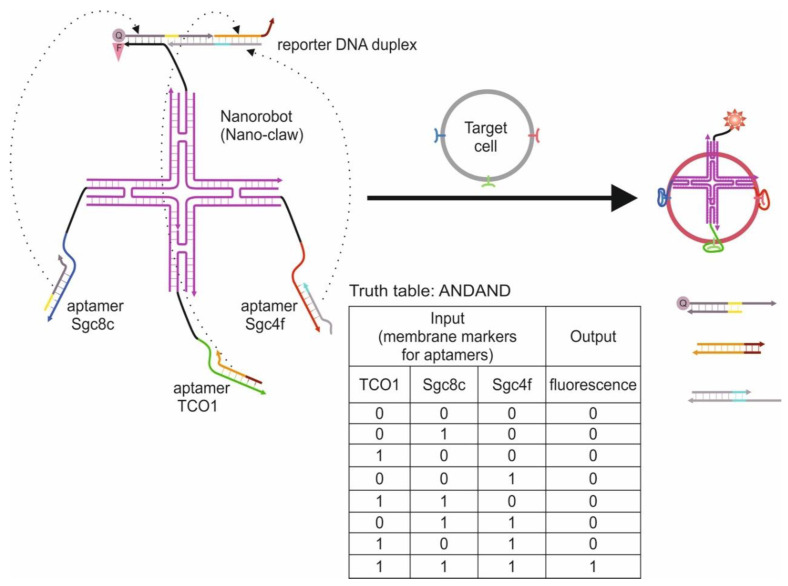
Logic gate i1ANDi2ANDi3. F = fluorescent label, Q = quencher label. Adapted from Reference [32]. Copyright © 2020, American Chemical Society.

A slightly different method of cell differentiation using a logical approach was proposed by Tan et al. in [32]. The aptamers were sgc8c (for membrane marker PTK7), sgc4f (exact target not established) and TC01 (exact target not established). The main essence of the considered approach was the use of logical robots (Nano-Claw) based on DNA with three legs in the form of a Y-structure and with four legs in the form of an X-structure (Figure 17). Structurally, the robot consisted of an oligonucleotide backbone and two (in the case of a Y-structure) or three (in the case of an X-structure) legs (“capture toe”) with aptamers and one DNA duplex as an analyzer of events with decision making (“effector toe”). The aptamers were directly bound to the oligonucleotide backbone and hybridized with small sequences (15 bp), which, upon binding of the aptamer to the target, were detached and interacted with dsDNA (“effector toe”). Interaction with the duplex was carried out through the substitution of the strand through the primers (toehold-mediated strand displacement reactions), while the cascade path was realized: the release of a new sequence initiated a downstream release reaction. Duplex DNA could contain a fluorescent label (in the duplex, the label was quenched by a nearby quencher) or a Ce6 photosensitive label for photodynamic therapy [156,157]. The result of the analyzer action was that the label was released from the quencher, thereby marking the cell containing certain membrane receptors. The output signal was fluorescence or cell viability assessment. The authors compiled the gate iANDi (contains two receptors), iINHIBITi (contains one receptor and does not contain the other) and iANDiANDi (contains three receptors). The first two gates were originally designed without using a nanorobot but using free aptamers with a complementary sequence and a tagged dsDNA that hybridized to the protruding end of the aptamer, thereby marking the cell that responded to the logic. However, in order to avoid possible false positive results from neighboring cells, aptamers began to be used as part of nanorobots, since aptamers are physically linked into one structure and are more likely to interact with the membrane proteins of one cell. It should be noted that despite an interesting design approach, the signal intensity from the cells was low, which somewhat complicates the diagnosis.

A logical aptameric sensor platform was developed to intelligently detect two types of cancer cells based on an inter-rod set of gold microelectrodes [33]. The essence of the method was that aptamers were immobilized on the surface of gold microelectrodes to capture target cells. If the target cells were in the sample, they were captured by the aptamers. Then, gold nanoparticles, also modified with aptamers against target cells, were added to the system to reduce the chip resistance, since they initially have a high electron transfer ability. Sgc8 against CCRF–CEM cells and TD05 against Ramos cells were used as aptamers. The change in the resistance of the microelectrodes modified by aptamers after the capture of target cells was the output signal of the system. The measurement was carried out by electrochemical impedance spectroscopy (EIS) using a redox pair [Fe(CN)_6_]^4−/3−^. In the absence of cells, the resistance between the electrodes was high (6.8–14.1 × 10^6^ ohms) and the circuit was considered to be open. Since the size of the cell was sufficient to close the circuit (the distance between the electrodes is 4–10 μm), the addition of cells to the modified electrodes led to the closure of the circuit and a decrease in the resistance between the electrodes. The OR and AND logic gates were developed with this in mind. At the same time, the design of the microelectrode structure for each valve was unique: located in parallel for OR and tandem for AND. Therefore, in the case of the OR gate, only if both types of target cells were absent (input (0, 0)), the microcircuit showed a high resistance (R > 10^5^ Ohm, output 0), corresponding to an open circuit. With any type of target cell (input (1.0), (0.1), or (1.1)), the gate resistance was reduced to <10^5^ ohms and the output was taken as 1. The OR gate can be used to determine the presence of target targets in the sample. For the AND gate, only in the presence of both types of target cells (input (1, 1)), the resistance of the entire microcircuit is significantly reduced (R < 10^5^ Ohm), which corresponds to a closed circuit, and the output of the AND gate was equal to 1. Thus, this logic gate can be used to check for both types of cancer cells in a sample.

Also, in continuation of previous studies, a DNA construct in the form of a prism was described for recognizing membrane targets of a cell and composing a logic AND gate [34]. The 3D prism, as with the previously described X- and Y-structures, has two legs with aptamers (“capture toe”) and one leg with one DNA duplex as an analyzer of events with decision making (“effector toe”). As before, aptamers are part of the 3D prism and hybridized with small DNA sequences, which, upon the interaction of the aptamer with the target, were detached and interacted with the DNA duplex (“effector toe”) through strand displacement through the primers. Due to the fact of this cascade, the quencher was separated from the fluorescent label directly connected to the prism. This allowed visualization of a cell containing targets for aptamers. Sgc8c and sgc4f were used as aptamers. It is worth noting that the use of a volumetric prism, as with the X- and Y-structures, allows one to minimize false positive signals from neighboring cells in comparison with unbound aptamers in logic [40]. It is worth noting that the level of expression of markers on the cell surface is also important: insufficient expression does not allow logic to be fully realized, which is shown by the example of Ramos cells and human acute lymphoblastic leukemia cells (CCRF–CEM).

**Figure 18:**
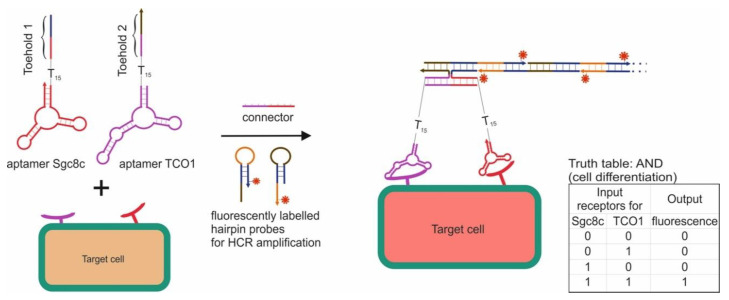
The AND logic gate. Adapted from Reference [35]. Copyright © 2020, American Chemical Society.

A method of discrimination of a certain type of cells in the general population of cells of a similar type has been shown [35]. Membrane proteins are excellent candidates for identifying cells of a certain subpopulation, since their set can be unique and represent a kind of molecular signature [158]. For more accurate identification of the cell type, it is required to carry out a multi-assay for a set of markers using logical instruments. The authors proposed AND logic based on anti-leukemia cell aptamers: sgc8c (against PTK7 receptor) [148,159] and TCO1 (against an unknown marker). There were three main components involved in the implementation of the logic gate. The first component is ssDNA, which contains a specific aptamer per membrane receptor, a spacer to eliminate steric restrictions, and a fragment for assembling a primer for a hybridization chain reaction (HCR). If both receptors are present on the cell surface, then ssDNA binds to them through aptamers, and each of them contains its own fragment of the primer which is the molecular barcode for the following operations. The second component is the ssDNA connector that hybridizes to overhanging DNA sequences to create a complete chain reaction primer. The connector serves as a linker to form an AND gate (Figure 18). The third component is two hairpins with a fluorescent Cy5 label each for the HCR. The HCR is a new class of non-enzymatic fluorescent signal amplification method for the detection of nucleic acids. In this procedure, the target DNA/RNA initiates a hybridization cascade between two fluorescently labeled hairpin sequences through toehold-mediated strand displacement [23]. The initiating DNA was a primer collected based on ssDNA with aptamers and an ssDNA connector. Thus, lymphoblastic leukemia cells containing both membrane receptors were fluorescently identified from a population of blood cancer cells. The incubation time of the cells with ssDNAs with the aptamers was 30 min, then, after washing, two other components were added and incubated for another 2 h before flow cytometry. Lymphoblastic leukemia cells were stained only in the presence of both aptamer-containing sequences (AND logic). Compared to other cancer cells, the fluorescent signal from lymphoblastic leukemia cells was 100 times higher, which indicates a high discrimination of the cell subpopulation among cells of a similar type. In addition, an AND gate was composed with three aptamers sgc4f, sgc8c, TCO1, i.e., recognizing three different membrane receptors. The proposed imaging method is sensitive and modular, allowing the identification of cells with different molecular signatures. In this case, the logic is implemented under mild conditions and with easily interpretable results (high or low fluorescence intensity). This is the first work combining several aptamers to carry out an HCR near the cell surface. The work which describes such a type of reaction near the cell surface with one aptamer has already been published [154].

## 5. Logic Gates Based on DNA Origami

The first appearance of DNA origami dates to 2006 [160]. DNA origami is a two- or three-dimensional nanostructure that is formed by directional interactions between a set of DNA complementary molecules.

Since DNA origami implies a modular approach to building structures, the aptamers are another module in the overall design of origami. The aptamers in DNA origami are partially hybridized with complementary DNA [36,37,161,162,163]. Molecular recognition of the target by aptamers is accompanied by conformational changes: switching of the aptamer from dsDNA to the complex with the target. As a result, aptamers can be used to activate the movement/action of DNA origami in response to the targets. In this case, the role of aptamers can be described as switchers. To date, several DNA origami structures have demonstrated the use of aptamers in the manner described above [161,164]. In this review, we will focus on the logic that can be implemented based on origami structures and aptamers. Some discussions can also be found in Reference [18].

Today, the main areas of use of DNA origami in combination with aptamers are medicine and pattern formation. In medicine, DNA origami is used in the form of nanocontainers or robots with cargo inside [36,37,163], while the aptamers serve as locks that open containers in the presence of a target—a key. These systems involve aptamers 41t [165], TE17 [166], sgc8c [159,167], AS1411 [168] against membrane targets of blood cells, usually cancer cells. The formation of patterns is also associated with the action of aptamers, which, when interacting with targets (ATP, cocaine), trigger either the assembly [161] or the disassembly [162] of DNA constructs. 

This section shows a logic gate based on a DNA nanorobot containing an internal cargo, with built-in aptamer locks to open it [36]; it also shows a system using a set of DNA nanorobots [37]. There is a use of a DNA frame with a logical controlled embedding of tiles inside the frame [161]. It is known a logical separation of the structure, consisting of several hexahedral DNA origami, linked by aptamers and complementary sequences [161].

**Figure 19:**
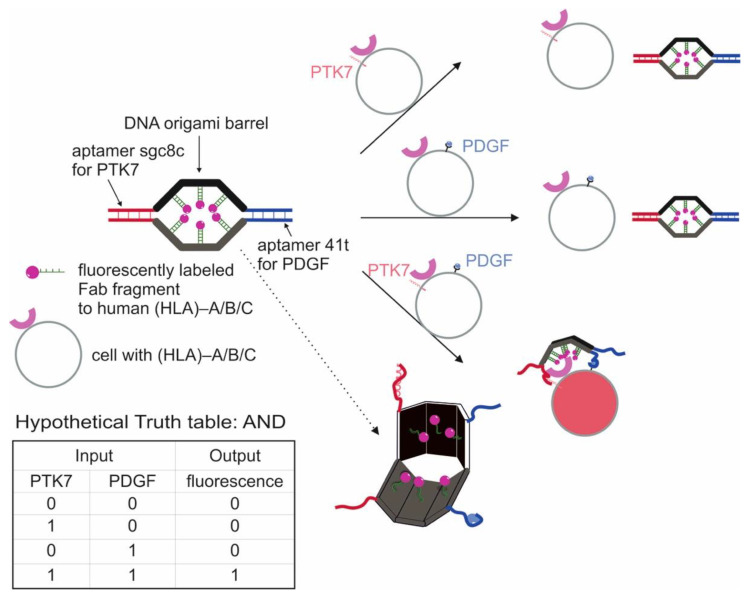
The AND logic gate. PDGF = platelet-derived growth factor. Adapted from Reference [36]. Copyright © 2020, Copyright © 2020, American Association for the Advancement of Science.

An important area of using logic on aptamers is the creation of DNA nanorobots (containers) capable of transporting a useful molecular cargo to cells [36]. Moreover, such DNA containers, due to the presence of aptamers in their structure, can recognize certain targets expressed by cells and open in this case, carrying out targeted delivery. The authors introduced the AND gate (Figure 19), when the container was opened only in the presence of two targets. Aptamers for proteins expressed by cancer blood cells were used as aptamers: 41t aptamer [165], TE17 [166], sgc8c [159,167]. Accordingly, the input data of the logic were membrane targets of these aptamers. The DNA container was assembled using the origami method [160] with the use of software for designing origami structures [169]. Self-assembly took place in one reaction vessel, in which 196 oligonucleotide “clips” guided the assembly of the 7308 bp phage filament with rapid annealing followed by slow cooling. The assembled structure was a barrel (35 nm × 35 nm × 45 nm), consisting of two domains covalently linked from one edge and with a lock on the other edge in the form of an aptamer duplex and a complementary sequence. In this case, the aptamer was located on one domain and the complementary sequence on the other. The optimal duplex length was 23 bp (activation at 10 pM target). The lock was opened when the aptamer was bound to the target, and the container was opened, freeing the cargo for further interaction. The cargo molecules were modified with a ssDNA linker which hybridized to the overhanging sequences within the container. To test the performance of the system, 5 nm gold nanoparticles and Fab fragments were used as weights. Next, nanorobots with different aptamer locks were loaded with fluorescently labeled antibodies against human leukocyte antigen (HLA)–A/B/C and mixed with various types of cancer cells expressing human HLA–A/B/C, as well as expressing various protein targets for aptamers in the lock. The mixture was incubated for 5 h. Using flow cytometry, the percentage of fluorescently labeled cells compared to the total cell amount was estimated. Also, the work of logic was evaluated in an experiment with a mixture of two types of cells: those that can open the container and those that cannot open it. The experiment showed that only cells expressing the protein target acquired the fluorescent label. This was also confirmed in experiments with healthy whole blood leukocytes. It should be noted that the accurate delivery of growth retardation factors for cancer leukocytes, as well as activation factors for T-cells, using such containers is shown. The proposed logic approach is extremely interesting from the point of view of targeted delivery and labeling.

**Figure 20:**
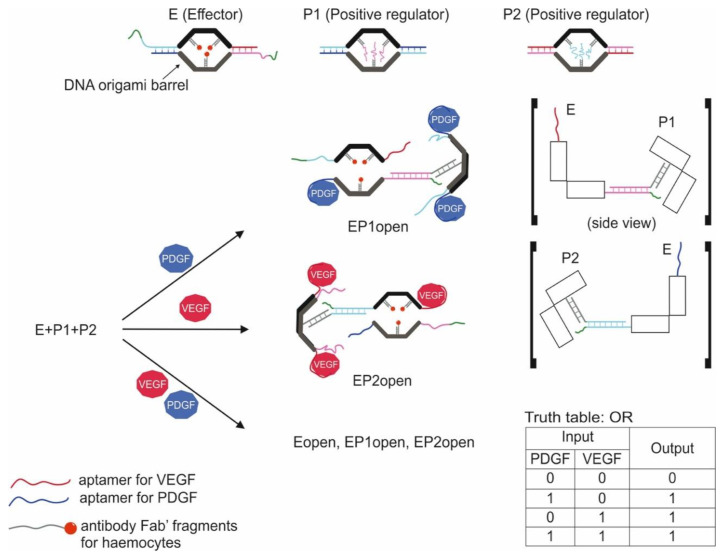
The OR logic gate. Adapted from Reference [37]. Copyright © 2020, Springer Nature.

Next, a series of logical operations AND, OR (Figure 20), XOR, NAND, NOT, CNOT, and half-adder was created based on the containers described above [36,37]. Aptamers on PDGF (41t) and VEGF were used as locks [170]. Thus, the input data were membrane targets for these aptamers. In this study, in addition to targets, the container can also be opened with an external DNA key (complementary sequence) using toehold-mediated strand displacement. Such a DNA key can be placed as a payload in one of the robots. Therefore, in the case when this nanorobot is open, the DNA key can gain access to the lock of a neighboring container. This corresponds to a “positive” type of regulator (P) loaded with an external key. A “negative” type of regulator (N) means that the nanorobot is loaded with DNA locks, which additionally lock two adjacent locks of a neighboring nanorobot, preventing it from opening. The payloads of the negative and positive regulators affected the effector nanorobot (E), which had two aptamer locks on PDGF and VEGF. The nanorobot effector was loaded with antibodies against insect hemocytes. Also, a nanorobot effector F with aptameric locks on PDGF and VEGF, which was not affected by “positive” and “negative” regulators, was used. Various logic gates were constructed by varying P, N, E, and F in the absence or presence of protein targets for aptamers. The output signal was a fluorescent signal from fluorescently labeled nanorobots using flow cytometry. The logic lasted several hours. This approach has been used ex vivo with live cockroaches *Blaberus discoidalis* for controlled logic and labeling of their cells. This work presents a new type of biological computing platform, the operation of which is suitable for controlling the precise delivery of drug molecules to living organisms. Since the nanorobot effector can be loaded with various drugs, and the regulators will open or block the effector in the event of a lack of any membrane target or an excess of both targets.

**Figure 21:**
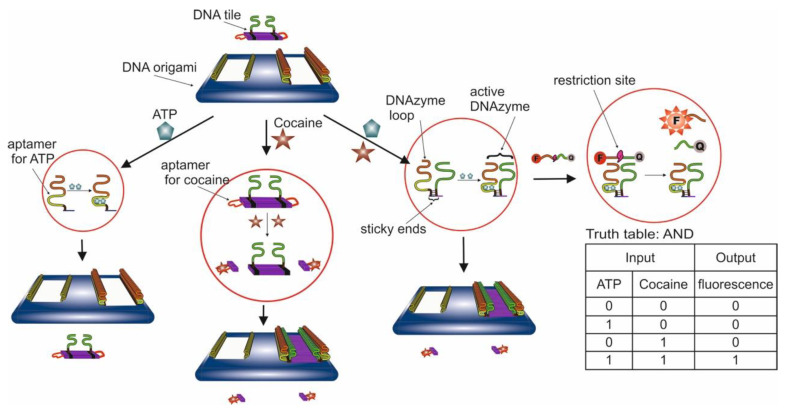
The AND logic gate. ATP = adenosine triphosphate, F = fluorescent label, Q = quencher label. Adapted from Reference [161]. Copyright © 2020, American Chemical Society.

In an interesting way the split strategy was used in the creation of logical YES, OR and AND gates based on separated DNAzyme and DNA origami structures [161]. The essence of the approach was that a rectangular DNA origami structure (~7000 bp circular ssDNA M13mp18 and 240 staples) had two holes that served as a frame for embedding DNA tiles into it. The insertion was due to the hybridization of the separated fragments of the magnesium-dependent DNAzyme E6 [171]: inside the holes on two opposite sides of DNA origami there were sticky ends (one of the DNAzyme fragments), one DNA tile contained four sticky ends (the second DNAzyme fragment), two on two opposite sides. The inner cavity of each hole can accommodate up to eight of these DNA tiles arranged in parallel. The sticky ends of the DNA tiles were blocked by an aptamer for ATP and cocaine. When ATP and cocaine were introduced into the system (input data of logic), the aptamers bound to the target and the DNA of the tile could be incorporated into the DNA origami frame. This is how the OR logic was implemented, with the presence of ATP in the system, one hole was filled, with the presence of cocaine, another. In this case, the output data were the data of atomic force microscopy and the fluorescent signal formed during the reaction of the collected DNAzyme with a fluorescent beacon. The YES logic was implemented using an external free DNAzyme E6, the catalytic core of which contained an aptamer on ATP and was inactive in the absence of a target. Upon the addition of ATP, the aptamer binds to the target and provides rigidity to the catalytic nucleus of DNAzyme [50] that became active. The active DNAzyme unwound the blocking sequence on the DNA tile, and the DNA tile was embedded in the frame. The embedding was detected by AFM (atomic force microscopy) and fluorescence from a split fluorescent beacon. The AND gate (Figure 21) was implemented by combining the approaches from the two previous ones: the introduction of cocaine led to the removal of the blocking sequence from the DNA of the tile, and the DNA tile was inserted into a frame with the assembly of inactive DNAzyme which was activated in the presence of ATP. ATP binds to the aptamer, which is part of one of the fragments of the assembled DNAzyme, which, thanks to this (allosteric regulation), acquired a rigid structure, and became active. Researchers show a programmable molecular logic operating system that generates a pattern on an origami DNA frame in a controlled manner. The time during which the logic was observed was 500 min. As the main drawback, the authors note the non-specific filling of the frame with origami tiles, which can be solved by more effective methods of protecting and releasing DNA fragments.

**Figure 22:**
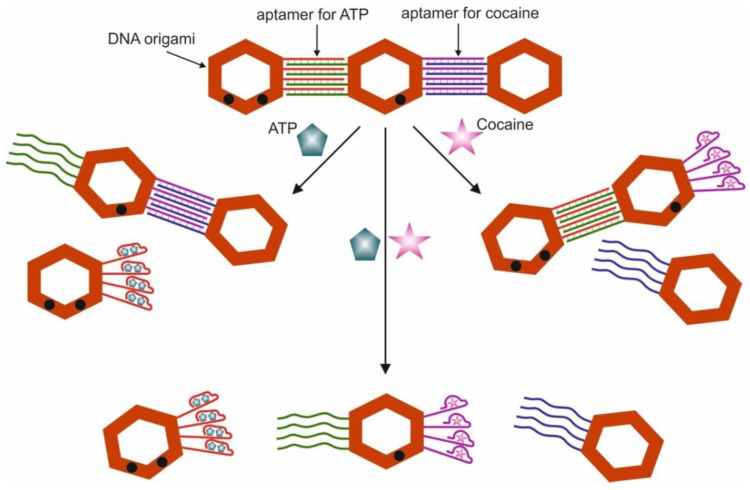
Logic scheme adapted from Reference [159] with permission from The Royal Society of Chemistry. ATP = adenosine triphosphate.

It is known the construction of logic using hexagonal origami structures and aptamers on ATP and cocaine [162]. Hexagonal DNA origami were obtained using M13 phage DNA and a set of staples for programmed folding to form a hollow hexagonal structure [172]. Each structure was linked to another to form dimers and trimers. The connection between origami DNA was carried out through the hybridization of four complementary DNAs located on one side of each origami. The DNA that is responsible for cross-linking included aptamers for ATP and cocaine. As a result, dimers and trimers were compiled. When the targets are introduced into the system, the composition composed of three structures is destroyed and dimers are formed if one target is added, and monomers are formed when two targets are added simultaneously (Figure 22). Thus, the trimer was obtained with a yield of 80%, which decreased to 19% in the presence of ATP or cocaine and decreased to 2.8% in the presence of both targets. The output signal was recorded using atomic force microscopy and PAGE electrophoresis. To distinguish each link in the DNA structure (trimmer), a streptavidin label was used. One DNA origami contained two tags, the other contained one tag, the next did not contain a tag. Thus, we can talk about the implementation of logic schemes based on aptamer and DNA origami. The logic implementation time was 120 min.

Recent work has demonstrated the successful use of a DNA origami-based nanorobot and a nucleolin aptamer for mammalian tumors, although without a direct logic gate [163]. The nanorobot was a sheet of DNA origami loaded with thrombin and then folded and closed with a nucleolin-binding aptamer. The nanorobot responded to one nucleolin input (YES gate); in the presence of nucleolin-positive tumor vascular endothelial cells, the container opened and released a payload, causing tumor vessel thrombosis with subsequent necrosis of tumor tissue, while maintaining healthy blood vessels.

## 6. Conclusions

In this review, we surveyed the different approaches to biomolecular computing realized with aptamers and different transducer platforms. At the moment, medicine and analytical chemistry can be considered the most promising and justified fields of its application. Since every year more and more new aptamers are obtained for various targets, therefore, the possibility of their application for the analysis of multi-component systems seems especially attractive. The concepts described in this review can serve as inspiration for the creation of new logic operations involving aptamers.

## Supplementary Materials

The following are available online at https://www.mdpi.com/1424-8247/13/11/417/s1, Table S1: Summary of logic gates based on aptamers (10.5281/zenodo.4049979).
