# Supplementary material for: Logic Gates Based on DNA Aptamers
Source: Pharmaceuticals (Basel). 2020 Nov 23;13(11):417. doi: 10.3390/ph13110417 (PMC7700249; doi:10.3390/ph13110417)
Supplement: Supplementary file 1 [file pharmaceuticals-13-00417-s001.pdf]

Table S1. Summary of logic gates based on aptamers.

Inputs (solid - targets for aptamers)	Logic gates	Aptameric strategy	Output signal	The operation time (from the moment of entering the input data to the moment of receiving the output signal), minutes	Reference
**Optical signal**					
**Fluorescent output in solution**					
Thrombin<br>Adenosine	iORi<br>iANDi	The use of bifunctional aptamer; switching of an aptamer from dsDNA to a complex with a target	Fluorescence (fluorescein)	30	[1]
Guanidinium chloride<br>prion PrP^{c}<br>prion PrP^{rev}	iXORi<br>iORi	Formation of a complex of an aptamer with a target	Fluorescence (quantum dots)	100	[2]
PDGF-BB<br>Hemin	iANDi	The use of bifunctional aptamer; switching of an aptamer from hairpin DNA to a complex with a target	Fluorescence (silver nanoclusters)	120	[3]
ATP<br>VEGF	iANDi	Switching of an aptamer from dsDNA to a complex with a target	Fluorescence (Texas Red, tetramethylrhodamine)	50	[4]
**The colorimetric output in the solution due to the catalytic labels**					
AMP<br>cocaine	iORi<br>iANDi<br>iXORi	Switching of an aptamer from dsDNA to a complex with a target	Colorimetric reaction of the hemin-containing DNAzyme	22	[5]
AMP<br>cocaine	iORi	The use of bifunctional aptamer; switching of an aptamer from dsDNA to a complex with a target	Colorimetric reaction of the hemin-containing DNAzyme	62	[6]
**Logic gates based on covalently modified gold nanoparticles**					
Hg^{2+} ions<br>*ssDNA*	iANDi<br>iORi<br>iAND/ORi<br>iNOTi	Formation of a complex of an aptamer with a target	Colorimetric signal from aggregation of AuNPs (SPR)	180	[7]
Adenosine<br>*ssDNA*	iXORi	Switching of an aptamer from dsDNA to a complex with a target	Fluorescence (Cy5)	Not defined	[8]

Adenosine<br>Cocaine<br>K^+ ions	iANDi<br>iORi	The use of bifunctional aptamer, switching of an aptamer from dsDNA to a complex with a target	Colorimetric signal from deaggregation of AuNPs (SPR)	5	[9]
VEGF<br>PDGF	iANDi<br>iORi	Switching of an aptamer from dsDNA to a complex with a target	OR: fluorescence (TAMRA, FITC)<br>AND: colorimetric signal from aggregation of AuNPs (SPR)	120	[10]
ATP<br>*ssDNA*	iANDiANDi	Switching of an aptamer from dsDNA to a complex with a target	Fluorescence (FAM)	17	[11]
**Lateral flow strip biosensors**					
ATP<br>Thrombine	iORi<br>iANDi	The use of a hybrid DNA based on cleaved and combined parts of aptamers; the use of split aptamers into two parts	Colorimetric signal from retaining of AuNPs (SPR)	45	[12]
Carcinoembryonic antigen (CEA)<br>*Aptamer against CEA*<br>*ssDNA*	iORi<br>iINHIBITi	Switching of an aptamer from hairpin DNA to a complex with a target	Colorimetric signal from retaining of AuNPs (SPR)	50 (OR)<br>80 (INHIBIT)	[13]
Thrombin<br>Mucin I<br>Carcinoembryonic antigen (CEA)<br>*Modified AuNPs*<br>*Aptamer against thrombin*<br>*Aptamer against mucin I*<br>*Aptamer against CEA*	iORi<br>iANDi<br>iINHIBITi<br>iNANDi<br>iANDiORi<br>iANDiINHIBITi<br>iORiINHIBITi<br>iINHIBITiNANDi<br>iANDiORiINHIBITi<br>iANDiINHIBITiNANDi<br>iORiINHIBITiNANDi<br>iANDiORiINHIBITiNANDi<br>keypad-lock system	Switching of an aptamer from hairpin DNA to a complex with a target; sandwich scheme (one target vs. two aptamers)	Colorimetric signal from retaining of AuNPs (SPR)	730	[14]
**Mesoporous silicon**					
Cocaine<br>Adenosine<br>K+ ions	iORi<br>iANDi	Switching of an aptamer from dsDNA to a complex with a target	Luminescence (rhodamine B)	70	[15]

Temperature					
**Logic gates based on unmodified gold nanoparticles**					
Adenosine<br>Cocaine	iANDi<br>iORi	The use of a hybrid DNA based on cleaved and combined parts of aptamers	Colorimetric signal from aggregation of AuNPs (SPR)	10	[16]
Bisphenol A<br>Bisphenol S<br>Aptamer against bisphenol A	iIMPLY1i<br>iIMPLY2i<br>iIMPLY1iIMPLY2i<br>iORi	Formation of a complex of an aptamer with a target	Colorimetric signal from aggregation of AuNPs (SPR)	18	[17]
D-arginine vasopressin<br>*Aptamer against D-arginine vasopressin*	iINHIBITi	Formation of a complex of an aptamer with a target	Colorimetric signal from aggregation of AuNPs (SPR)	40	[18]
D-arginin<br>L-arginin	iORi	Formation of a complex of an aptamer with a target	Colorimetric signal from aggregation of AuNPs (SPR)	45	[19]
**Hydrogels**					
ATP<br>cocaine	iORi<br>iANDi	The use of a bifunctional aptamer; switching of an aptamer from dsDNA to a complex with a target	Colorimetric signal from release of AuNPs (SPR)	25	[20]
Hg^{2+} ions<br>Ag^{+} ions	iORi<br>iANDi	Formation of a complex of an aptamer with a target	Colorimetric signal from SiNPs (structural color)	Not defined	[21]
**Logical gates based on graphene oxide**					
ATP<br>Thrombin<br>*Aptamer against thrombin*<br>*Aptamer against ATP*	iINHIBITi<br>iORi	Switching of an aptamer from grapheme surface to a complex with a target	Fluorescence (FAM)	30	[22]
ATP<br>Thrombin	iANDi	Formation of a complex of an aptamer with a target	Fluorescence (FAM, ROX)	150 (time of fluorescence recording)	[23]
Thrombin<br>PDGF-BB<br>*ssDNA*	iORi<br>iINHIBITi	Switching of an aptamer from grapheme surface to a complex with a target; sandwich scheme (one target vs. two aptamers)	Colorimetric signal from gemin-containing graphene	102	[24]
ATP<br>Thrombin	half-adder<br>half-substractor	Switching of an aptamer from grapheme surface to a	Fluorescence (QD_{506} и QD_{571})	40	[25]

Aptamer against thrombin<br>*Aptamer against ATP*		complex with a target			
ATP<br>ssDNA	iANDi	Switching of an aptamer from dsDNA to a complex with a target	Fluorescence (PicoGreen dye for dsDNA)	180	[26]
**Elecrtochemical detection**					
**Two-/three electrode cells**					
**Electroactive compounds**					
Cocaine<br>*ssDNA*	iXORi	Formation of a complex of an aptamer with a target	Change in the reduction current (methylene blue)	Not defined	[27]
ATP<br>*Adenosine deaminase*<br>*$K_3[Fe(CN)_6]$*<br>*K_2IrCl_6*	iINHIBITi<br>iINHIBITiANDi<br>iINHIBITiANDiXORi	The use of split aptamer into two parts	Change in the current (ferrocene)	>30	[28]
Thrombin<br>Lysozyme	iNANDi	Switching of an aptamer from graphene electrode surface to a complex with a target; the use of bifunctional aptamer	Change in the current ($[Ru(NH_3)_6]^{3+}$)	60	[29]
**Catalytic label**					
Kanamycin<br>Oxytetracycline<br>*Aptamer against kanamycin*<br>*Aptamer against oxytetracycline*	iORi<br>iINHIBITi	The use of bifunctional aptamer	Chronopotentiometric detection (hemin-containing DNAzyme reaction)	60	[30]
Thrombin<br>ATP	iORi<br>iANDi<br>iNORi<br>iNANDi	The use of a hybrid DNA based on cleaved and combined parts of aptamers; The use of split aptamer into two parts; switching of an aptamer from dsDNA to a complex with a target	Reduction current (HRP)	88	[31]
**Biofuel cells**					
Thrombin<br>Lysozyme	iNANDi	Formation of a complex of an aptamer with a target	Open circuit voltage	Not defined	[32]
Thrombin	ON-OFF	The use of bifunctional	Current density on the	120	[33]

ATP	RESET	aptamer; switching of an aptamer from dsDNA to a complex with a target	cathode		
ATP<br>*Adenosine deaminase*	iINHIBITi	Switching of an aptamer from hairpin DNA to a complex with a target	Cathode current	120	[34]
		**Other**			
ATP<br>*ssDNA*	IMPLICATION (if..then)<br>ON-OFF	Switching of an aptamer from dsDNA to a complex with a target	Ion current	500 (ON/OFF)<br>100 (OFF/ON).	[35]
		**Cell-based logic gates**			
Membrane targets for aptamers<br>sgc8c,<br>TD05,<br>sgc4f,<br>TE17,<br>TE02	i1ANDi2<br>i1ORi2<br>i1NOTi2<br>i1ANDNOTi2 i1ANDi2ANDi3<br>i1AND(i2ORi3)<br>i1ANDNOT(i2ORi3)<br>i1ANDi2ANDNOTi3<br>i1ANDi2ANDi3ANDi4<br>i1ANDi2ANDi3ANDNOTi4	Switching of an aptamer from dsDNA to a complex with a target	Fluorescence (cytometry), assessment of cell viability (propidium iodide)	>90	[36]
Membrane targets for aptamers<br>sgc8c<br>sgc4f<br>TC01	iANDi<br>iINHIBITi<br>iANDiANDi	Switching of an aptamer from dsDNA to a complex with a target	Fluorescence (cytometry), assessment of cell viability (propidium iodide)	>90	[37]
Membrane targets for aptamers<br>sgc8c<br>TD05	iORi<br>iANDi	Formation of a complex of an aptamer with a target	The change of resistance between electrodes	Not defined	[38]
Membrane targets for aptamers<br>sgc8c<br>sgc4f	iANDi	Switching of an aptamer from dsDNA to a complex with a target	Fluorescence (cytometry)	60-240	[39]
Membrane targets for aptamers<br>sgc8c<br>sgc4f<br>TC01	iANDi	Formation of a complex of an aptamer with a target	Fluorescence (cytometry)	150	[40]
		**Logic gates based on DNA origami**			
Membrane targets for	iANDi	Switching of an aptamer from	Fluorescence	>300	[41]

aptamers<br>41t<br>TE17<br>sgc8c		dsDNA to a complex with a target	(cytometry)		
Membrane targets for aptamers<br>PDGF<br>VEGF	iANDi<br>iORi<br>iXORi<br>iNANDi<br>iNOTi<br>iCNOTi	Switching of an aptamer from dsDNA to a complex with a target	Fluorescence (cytometry)	Several hours	[42]
ATP<br>Cocaine	YES<br>iORi<br>iANDi	Switching of an aptamer from dsDNA to a complex with a target; formation of a complex of an aptamer with a target	Atomic force microscopy, fluorescence (cleaving DNAzyme)	500	[43]
ATP<br>Cocaine	Conditionally<br>iORi<br>iANDi	Switching of an aptamer from dsDNA to a complex with a target	Atomic force microscopy, PAGE electrophoresis	120	[44]

References

1. Yoshida, W.; Yokobayashi, Y. Photonic boolean logic gates based on DNA aptamers. *Chem. Commun.* **2007**, 195–197.
2. Xiao, S.J.; Hu, P.P.; Chen, L.Q.; Zhen, S.J.; Peng, L.; Li, Y.F.; Huang, C.Z. A Visual Dual-Aptamer Logic Gate for Sensitive Discrimination of Prion Diseases-Associated Isoform with Reusable Magnetic Microparticles and Fluorescence Quantum Dots. *PLoS One* **2013**, *8*.
3. Wang, G.; Zhu, Y.; Chen, L.; Zhang, X. Photoinduced electron transfer (PET) based label-free aptasensor for platelet-derived growth factor-BB and its logic gate application. *Biosens. Bioelectron.* **2015**, *63*, 552–557.
4. Liao, W.C.; Sohn, Y.S.; Riutin, M.; Cecconello, A.; Parak, W.J.; Nechushtai, R.; Willner, I. The Application of Stimuli-Responsive VEGF- and ATP-Aptamer-Based Microcapsules for the Controlled Release of an Anticancer Drug, and the Selective Targeted Cytotoxicity toward Cancer Cells. *Adv. Funct. Mater.* **2016**, *26*, 4262–4273.
5. Shlyahovsky, B.; Li, Y.; Lioubashevski, O.; Elbaz, J.; Willner, I. Logic gates and antisense DNA devices operating on a translator nucleic acid scaffold. *ACS Nano* **2009**, *3*, 1831–1843.
6. Elbaz, J.; Shlyahovsky, B.; Li, D.; Willner, I. Parallel analysis of two analytes in solutions or on surfaces by using a bifunctional aptamer: Applications for biosensing and logic gate operations. *ChemBioChem* **2008**, *9*, 232–239.
7. Ogawa, A.; Susaki, Y. Multiple-input and visible-output logic gates using signal-converting DNA machines and gold nanoparticle aggregation. *Org. Biomol. Chem.* **2013**, *11*, 3272–3276.
8. Liu, J.; Ji, H.; Huang, J.; Li, L.; Wang, Q.; Yang, X.; Wang, K. Intelligent Nucleic Acid Functionalized Dual-Responsive Gold Nanoflare: Logic-Gate Nanodevice Visualized by Single-Nanoparticle Imaging. *ChemistrySelect* **2016**, *1*, 347–353.

9. Liu, J.; Lu, Y. Smart nanomaterials responsive to multiple chemical stimuli with controllable cooperativity. *Adv. Mater.* **2006**, *18*, 1667–1671.
10. Shukoor, M.I.; Altman, M.O.; Han, D.; Bayrac, A.T.; Ocsoy, I.; Zhu, Z.; Tan, W. Aptamer-nanoparticle assembly for logic-based detection. *ACS Appl. Mater. Interfaces* **2012**, *4*, 3007–3011.
11. Yang, B.; Zhang, X.B.; Kang, L.P.; Huang, Z.M.; Shen, G.L.; Yu, R.Q.; Tan, W. Intelligent layered nanoflare: "Lab-on-a-nanoparticle" for multiple DNA logic gate operations and efficient intracellular delivery. *Nanoscale* **2014**, *6*, 8990–8996.
12. Chen, J.; Fang, Z.; Lie, P.; Zeng, L. Computational lateral flow biosensor for proteins and small molecules: A new class of strip logic gates. *Anal. Chem.* **2012**, *84*, 6321–6325.
13. Huang, Y.; Wen, W.; Du, D.; Zhang, X.; Wang, S.; Lin, Y. A universal lateral flow biosensor for proteins and DNAs based on the conformational change of hairpin oligonucleotide and its use for logic gate operations. *Biosens. Bioelectron.* **2014**, *61*, 598–604.
14. Qin, C.; Gao, Y.; Wen, W.; Zhang, X.; Wang, S. Visual multiple recognition of protein biomarkers based on an array of aptamer modified gold nanoparticles in biocomputing to strip biosensor logic operations. *Biosens. Bioelectron.* **2016**, *79*, 522–530.
15. Wen, Y.; Xu, L.; Li, C.; Du, H.; Chen, L.; Su, B.; Zhang, Z.; Zhang, X.; Song, Y. DNA-based intelligent logic controlled release systems. *Chem. Commun.* **2012**, *48*, 8410–8412.
16. Xu, X.; Zhang, J.; Yang, F.; Yang, X. Colorimetric logic gates for small molecules using split/integrated aptamers and unmodified gold nanoparticles. *Chem. Commun.* **2011**, *47*, 9435–9437.
17. Ragavan, K. V.; Selvakumar, L.S.; Thakur, M.S. Functionalized aptamers as nano-bioprobes for ultrasensitive detection of bisphenol-A. *Chem. Commun.* **2013**, *49*, 5960–5962.
18. Ren, J.; Wang, J.; Wang, J.; Wang, E. Colorimetric enantiorecognition of oligopeptide and logic gate construction based on DNA aptamer-ligand-gold nanoparticle interactions. *Chem. - A Eur. J.* **2013**, *19*, 479–483.
19. Yuan, H.; Huang, Y.; Yang, J.; Guo, Y.; Zeng, X.; Zhou, S.; Cheng, J.; Zhang, Y. An aptamer-based fluorescence bio-sensor for chiral recognition of arginine enantiomers. *Spectrochim. Acta - Part A Mol. Biomol. Spectrosc.* **2018**, *200*, 330–338.
20. Yin, B.C.; Ye, B.C.; Wang, H.; Zhu, Z.; Tan, W. Colorimetric logic gates based on aptamer-crosslinked hydrogels. *Chem. Commun.* **2012**, *48*, 1248–1250.
21. Ye, B.; Wang, H.; Ding, H.; Zhao, Y.; Pu, Y.; Gu, Z. Colorimetric logic response based on aptamer functionalized colloidal crystal hydrogels. *Nanoscale* **2015**, *7*, 7565–7568.
22. Wang, L.; Zhu, J.; Han, L.; Jin, L.; Zhu, C.; Wang, E.; Dong, S. Graphene-based aptamer logic gates and their application to multiplex detection. *ACS Nano* **2012**, *6*, 6659–6666.
23. Liu, X.; Aizen, R.; Freeman, R.; Yehezkeli, O.; Willner, I. Multiplexed aptasensors and amplified dna sensors using functionalized graphene oxide: Application for logic gate operations. *ACS Nano* **2012**, *6*, 3553–3563.
24. Lin, B.; Sun, Q.; Liu, K.; Lu, D.; Fu, Y.; Xu, Z.; Zhang, W. Label-free colorimetric protein assay and logic gates design based on the self-assembly of hemin-graphene hybrid nanosheet. *Langmuir* **2014**, *30*, 2144–2151.
25. Hu, X.; Liu, Y.; Qu, X.; Sun, Q. A quantum dot-labelled aptamer/graphene oxide system for the construction of a half-adder and half-subtractor with high resettability. *Chem. Commun.* **2017**, *53*, 11181–11184.
26. Zhang, J.; Yang, C.; Niu, C.; Liu, C.; Cai, X.; Du, J.; Chen, Y. A label-free fluorescent AND logic gate aptasensor for sensitive ATP detection. *Sensors (Switzerland)* **2018**, *18*.

27. Xia, F.; Zuo, X.; Yang, R.; White, R.J.; Xiao, Y.; Kang, D.; Gong, X.; Lubin, A.A.; Vallée-Bélisle, A.; Yuen, J.D.; et al. Label-free, dual-analyte electrochemical biosensors: A new class of molecular-electronic logic gates. *J. Am. Chem. Soc.* **2010**, *132*, 8557–8559.
28. Feng, L.; Lyu, Z.; Offenhäusser, A.; Mayer, D. Multi-level logic gate operation based on amplified aptasensor performance. *Angew. Chemie - Int. Ed.* **2015**, *54*, 7693–7697.
29. Du, M.; Yang, T.; Zhao, C.; Jiao, K. Electrochemical logic aptasensor based on graphene. *Sensors Actuators, B Chem.* **2012**, *169*, 255–260.
30. Liu, S.; Ding, J.; Qin, W. Dual-Analyte Chronopotentiometric Aptasensing Platform Based on a G-Quadruplex/Hemin DNAzyme and Logic Gate Operations. *Anal. Chem.* **2019**, *91*, 3170–3176.
31. Chen, J.; Zeng, L. Enzyme-amplified electronic logic gates based on split/intact aptamers. *Biosens. Bioelectron.* **2013**, *42*, 93–99.
32. Zhou, M.; Du, Y.; Chen, C.; Li, B.; Wen, D.; Dong, S.; Wang, E. Aptamer-controlled biofuel cells in logic systems and used as self-powered and intelligent logic aptasensors. *J. Am. Chem. Soc.* **2010**, *132*, 2172–2174.
33. Zhou, M.; Chen, C.; Du, Y.; Li, B.; Wen, D.; Dong, S.; Wang, E. An IMP-Reset gate-based reusable and self-powered "smart" logic aptasensor on a microfluidic biofuel cell. *Lab Chip* **2010**, *10*, 2932–2936.
34. Zhou, M.; Kuralay, F.; Windmiller, J.R.; Wang, J. DNAzyme logic-controlled biofuel cells for self-powered biosensors. *Chem. Commun.* **2012**, *48*, 3815–3817.
35. Jiang, Y.; Liu, N.; Guo, W.; Xia, F.; Jiang, L. Highly-efficient gating of solid-state nanochannels by DNA supersandwich structure containing ATP aptamers: A nanofluidic IMPLICATION logic device. *J. Am. Chem. Soc.* **2012**, *134*, 15395–15401.
36. You, M.; Zhu, G.; Chen, T.; Donovan, M.J.; Tan, W. Programmable and multiparameter DNA-based logic platform for cancer recognition and targeted therapy. *J. Am. Chem. Soc.* **2015**, *137*, 667–674.
37. You, M.; Peng, L.; Shao, N.; Zhang, L.; Qiu, L.; Cui, C.; Tan, W. DNA "nano-claw": Logic-based autonomous cancer targeting and therapy. *J. Am. Chem. Soc.* **2014**, *136*, 1256–1259.
38. Lou, B.; Zhou, Z.; Du, Y.; Dong, S. Resistance-based logic aptamer sensor for CCRF-CEM and Ramos cells integrated on microfluidic chip. *Electrochem. commun.* **2015**, *59*, 64–67.
39. Peng, R.; Zheng, X.; Lyu, Y.; Xu, L.; Zhang, X.; Ke, G.; Liu, Q.; You, C.; Huan, S.; Tan, W. Engineering a 3D DNA-Logic Gate Nanomachine for Bispecific Recognition and Computing on Target Cell Surfaces. *J. Am. Chem. Soc.* **2018**, *140*, 9793–9796.
40. Chang, X.; Zhang, C.; Lv, C.; Sun, Y.; Zhang, M.; Zhao, Y.; Yang, L.; Han, D.; Tan, W. Construction of a Multiple-Aptamer-Based DNA Logic Device on Live Cell Membranes via Associative Toehold Activation for Accurate Cancer Cell Identification. *J. Am. Chem. Soc.* **2019**, *141*, 12738–12743.
41. Douglas, S.M.; Bachelet, I.; Church, G.M. A logic-gated nanorobot for targeted transport of molecular payloads. *Science (80-.).* **2012**, *335*, 831–834.
42. Amir, Y.; Ben-Ishay, E.; Levner, D.; Ittah, S.; Abu-Horowitz, A.; Bachelet, I. Universal computing by DNA origami robots in a living animal. *Nat. Nanotechnol.* **2014**, *9*, 353–357.
43. Yang, J.; Jiang, S.; Liu, X.; Pan, L.; Zhang, C. Aptamer-Binding Directed DNA Origami Pattern for Logic Gates. *ACS Appl. Mater. Interfaces* **2016**, *8*, 34054–34060.
44. Wu, N.; Willner, I. Programmed dissociation of dimer and trimer origami structures by aptamer-ligand complexes. *Nanoscale* **2017**, *9*, 1416–1422.
